# Social Isolation Changes and Long-Term Outcomes Among Older Adults

**DOI:** 10.1001/jamanetworkopen.2024.24519

**Published:** 2024-07-24

**Authors:** Chen Lyu, Katherine Siu, Ian Xu, Iman Osman, Judy Zhong

**Affiliations:** 1 Division of Biostatistics, Department of Population Health, New York University Langone Medical Center, New York; 2 Department of Medicine, New York University Langone Medical Center, New York; 3 Ronald Perlman Department of Dermatology, New York University Langone Medical Center, New York

## Abstract

**Importance:**

While the association between cross-sectional measures of social isolation and adverse health outcomes is well established, less is known about the association between changes in social isolation and health outcomes.

**Objective:**

To assess changes of social isolation and mortality, physical function, cognitive function, cardiovascular disease (CVD), and stroke.

**Design, Setting, and Participants:**

In a cohort design, social isolation changes in 4 years and subsequent risk of mortality and other outcomes were assessed using the 13 649 eligible Health and Retirement Study (HRS) respondents from the 2006 to 2020 waves. Data were analyzed from October 11, 2023, to April 26, 2024.

**Exposure:**

The main exposure was the change in social isolation measured by the Steptoe 5-item Social Isolation Index from the initial assessment to a second assessment conducted 4 years later. Participants were classified into decreased isolation, stable, or increased isolation groups, stratified by their baseline isolation status.

**Main Outcomes and Measures:**

The primary outcomes were mortality, self-reported dependencies in activities of daily living, Alzheimer disease and Alzheimer disease–related dementia, CVD, and stroke. Dementia, CVD, and stroke were assessed using HRS-linked Medicare records. Incidence rates (IRs) of each group were estimated and a Cox proportional hazards regression model was used, with inverse-probability treatment weighting to adjust for confounders.

**Results:**

Among 13 649 participants (mean [SD] age at baseline, 65.3 [9.5] years; 8011 [58.7%] women) isolated at baseline, those with increased isolation had higher mortality (n = 693; IR = 68.19; 95% CI, 60.89-76.36 per 1000 person-years) than those who were stable (n = 1796; IR = 44.02; 95% CI, 40.47-47.88 person-years) or had decreased isolation (n = 2067; IR = 37.77; 95% CI, 34.73-41.09 person-years) isolation. Increased isolation was associated with higher risks of mortality (adjusted hazard ratio [AHR], 1.29; 95% CI, 1.09-1.51), disability (AHR, 1.35; 95% CI, 1.09-1.67), and dementia (AHR, 1.40; 95% CI, 1.02-1.93) compared with stable isolation. Similar findings were observed among socially nonisolated participants at baseline.

**Conclusions and Relevance:**

In this cohort study, increased isolation was associated with elevated risks of mortality, disability, and dementia, irrespective of baseline isolation status. These results underscore the importance of interventions targeting the prevention of increased isolation among older adults to mitigate its adverse effects on mortality, as well as physical and cognitive function decline.

## Key Points

**Question:**

Is social isolation change associated with long-term outcomes in older adults?

**Findings:**

In this cohort study using a national longitudinal health survey of 13 649 adults aged 50 years or older in the US, data revealed that increased isolation was associated with an increased risk of mortality, disability, and dementia. Decreased isolation was associated with a lower risk of mortality only among individuals who were nonisolated at baseline.

**Meaning:**

These results underscore the importance of interventions targeting the prevention of increased isolation among older adults to mitigate its adverse effects on mortality, as well as physical and cognitive function decline.

## Teaser

This cohort study examines the long-term health outcomes associated with changes in social isolation status among individuals aged 50 years or older participating in the Health and Retirement Study.

## Introduction

Social isolation manifests as an objective and measurable consequence characterized by a diminished social network size and a shortage of social interactions.^1,2,3,4^ The increasing prevalence of social isolation among older adults has become a focal point in health and social policy discussions, raising major concerns.^5,6,7^ Previous research consistently underscores the heightened vulnerability of older adults to social isolation.^4,6,7,8^ An estimated 20% to 25% of community-dwelling older adults are categorized as socially isolated, including 4% experiencing severe social isolation.^4,7,9^ This is particularly important, as social isolation has been identified as a risk factor contributing to a range of adverse health consequences, including increasing mortality,^2,10,11^ cognitive health decline,^12^ heart disease,^13,14,15^ and decreasing physical activities.^8,10,11,16,17,18,19,20,21^

The dynamic nature of mental health undergoes constant change. However, much of the existing research on social isolation relies on cross-sectional measurements, overlooking the association between changes in social isolation and subsequent health outcomes. While a few studies have reported associations, such as increased social isolation was associated with functional limitations and memory decline,^22,23^ this area remains underevaluated. This gap in knowledge impedes our ability to assess interventions aimed at preventing increased isolation or promoting social connections to improve health outcomes.

In this study, we estimated the association between social isolation change and risks of mortality and other health outcomes, including disability, dementia, cardiovascular disease (CVD), and stroke, using the Health and Retirement Study (HRS), a longitudinal, population-based survey study of older adults.^24^ We analyzed the associations in individuals with and without baseline social isolation. To our knowledge, this study represents the largest of its kind to estimate the association between social isolation changes and health outcomes stratified by baseline isolation status.

## Methods

### Data and Study Design

We analyzed data from 6 birth cohorts of HRS participants from the 2006 to 2020 waves.^24^ The HRS is a nationally representative, biennial, longitudinal health interview survey of adults aged 50 years and older in the US.^24,25^ All respondents provide informed consent and receive token payment on their entry into the HRS. The HRS is sponsored by the National Institute on Aging and is performed by the Institute for Social Research at the University of Michigan, Ann Arbor; it has been approved by the University of Michigan Health Sciences Institutional Review Board. The data used in this analysis are retrieved from RAND HRS^26^ and contain no unique identifiers. This study followed the Strengthening the Reporting of Observational Studies in Epidemiology (STROBE) reporting guideline. Data were analyzed from October 11, 2023, to April 26, 2024.

### Variables and Measurements

#### Social Isolation Exposure

Leave-Behind Questionnaires (LBQ) were included in the HRS biennial core survey starting from 2006-2008, which included the Steptoe 5-item Social Isolation Index (SII) to measure social isolation status.^27,28^ With SII, each respondent was assigned a positive response to each item, including if they (1) were unmarried/living alone, (2) had less than monthly contact with children (all contacts, including face-to-face meet up, speak on the phone, write, or email), (3) had less than monthly contact with other family members, (4) had less than monthly contact with friends, and (5) did not participate monthly in any groups, clubs, or other social organizations. The final score of the SII was the sum of the 5 items, ranging from 0 to 5, with higher scores indicating more isolated status. Respondents scoring 2 or above were categorized as socially isolated.^9,16^ Numerous studies have confirmed the reliability and validity of SII use for older adults.^16,29^

Alternatively, half of HRS respondents were chosen for LBQ, resulting in their assessments of SII every 4 years. We defined each respondent’s baseline assessment as the year of their initial SII measurement, with the second SII measurement occurring 4 years after baseline. The primary exposure was the change in the SII score from the initial baseline assessment to the second measurement. Respondents were then categorized as (1) decreased isolation if the score of their second measurement decreased by 1 unit or more from baseline, (2) stable if the score of their second measurement remained unchanged from baseline, and (3) increased isolation if the score of their second measurement increased by 1 unit or more from baseline.

For our sensitivity analysis, we used an alternative approach, defining changes in social isolation based on binary isolation status transitions. Specifically, for individuals initially isolated, we categorized change groups as transitioning from isolation to nonisolation vs remaining isolated. Conversely, for initially nonisolated individuals, change groups were defined as transitioning from nonisolation to isolation vs remaining nonisolated.

#### Outcomes

We investigated 5 incident outcomes: mortality, disability, dementia, CVD, and stroke. The starting time for all outcomes was defined as the second SII measurement, 4 years after baseline. The time of the event was defined as the earliest occurrence of an event after the starting time (second SII measurement). Death and the year of death in the HRS were confirmed using the National Death Index^30^ and the Social Security Death Index.^31^ Disability was assessed in the HRS core survey through self-reported activities of daily living (ADLs)^32^ dependencies (walking across a room, dressing, bathing, eating, getting in or out of bed, and using the toilet) exceeding 0. The year of incident disability onset was estimated as the first year when a respondent reported at least 1 ADL dependency. Participants with no events for death or disability were censored at their last HRS interview up to 2020. Dementia diagnosis was retrieved from HRS-linked Medicare records (1991-January 1, 2017), categorized by the Medicare Chronic Conditions Data Warehouse (CCW)^33^ for the disease categories Alzheimer disease or Alzheimer disease–related dementia. Similarly, CVD diagnosis was retrieved for disease categories in the CCW including acute myocardial infarction, atrial fibrillation, congestive heart failure, or ischemic heart disease. Stroke diagnosis was retrieved from the CCW for the disease category stroke. Detailed *International Statistical Classification of Diseases and Related Health Problems, 10th Revision* codes for each category were provided by the Master Beneficiary Summary File and are listed in eTable 1 in Supplement 1. The year of onset of each outcome was defined as the earliest year when a respondent had such a claim, with participants having no such claims censored to the last date of linked Medicare data (January 1, 2017).

For the mortality outcome, our analysis cohort comprised HRS respondents from the 2006 to 2020 waves, aged 50 years or older at baseline, with nonmissing baseline SII measurements and nonmissing second SII measurements 4 years after baseline. For other outcomes, eligible samples were further limited to the same analysis cohort with linked Medicare records and without the outcome of interest at baseline (first SII measurement) or at the starting time (second SII measurement). Each outcome was analyzed independently.

#### Covariates

Demographic variables included age, sex, self-reported race and ethnicity (Hispanic, non-Hispanic Black, non-Hispanic White, and Other [American Indian, Alaskan Native, Asian, Native Hawaiian and Pacific Islander, and other race or unknown]), baseline year, HRS birth cohort, self-respondent vs proxy, educational attainment, total assets in quantile, and marital or partner status. Because different racial and ethnic groups may experience varying levels of social isolation and its associated health effects due to socioeconomic factors, cultural differences, and historical contexts, race and ethnicity was included in the analysis to ensure that demographic diversity and potential disparities in social isolation and health outcomes were accounted for. Clinical variables included body mass index, smoking, Center for Epidemiological Studies–Depression (CES-D) score, ADL score, model-adjusted 27-point cognition score,^34^ and comorbid conditions (self-reported hypertension, diabetes, lung disease, heart disease, stroke, cancer, psychiatric problem, and arthritis), all assessed at baseline and their change from baseline to the second SII measurement. Baseline SII was also adjusted as a covariate.

### Statistical Analysis

We first performed descriptive analyses to characterize the groups of social isolation change. Descriptive comparisons were performed using analysis of variance or a Wilcoxon rank sum test for continuous measures and χ^2^ test for categorical measures. Cumulative incidence curves and incidence rates (IRs) of each outcome were estimated. Time-to-event was defined from the year of the second SII measurement to the time of event if the participants had the event or to the time of the last HRS interview if the participants did not have the outcome. Death was treated as a competing outcome for disability, dementia, CVD, and stroke, analyzed by the Fine-Gray model.

To mitigate potential confounding effects, we used inverse probability for treatment weights (IPTW).^35^ To calculate the denominator of the IPTW, we used multinomial logistic regression models to model the 3 groups as a function of both baseline covariates and changes in covariates between baseline and the second SII measurement.^35^ We included the same covariates as potential confounders regardless of their significance in the multinomial model. These baseline covariates included age, sex, HRS cohort, race and ethnicity, educational level, total assets, marital status, body mass index, smoking status, CES-D scores, ADL dependence, cognition, and comorbidities; the change in covariates included changes in CES-D, ADL, cognition, and comorbidities from baseline to the second SII measurement. To assess the IPTW, we conducted a comparison between the absolute standardized differences in covariates for the unweighted and weighted samples. Following that, we used IPTW-weighted Cox proportional hazards regression models to analyze each time-to-event outcome, adjusting for all covariates. Separate analysis was performed for baseline nonisolated and isolated groups.

We performed 2 additional sensitivity analyses. First, we excluded individuals who died within 2 years after the second SII measurement. Second, we incorporated HRS sampling weights at baseline. All analyses were performed in R, version 4.3.1, with package IPTW (R Project for Statistical Computing). Significance was defined as *P* < .05, using 2-sided tests.

## Results

**Figure 1.**
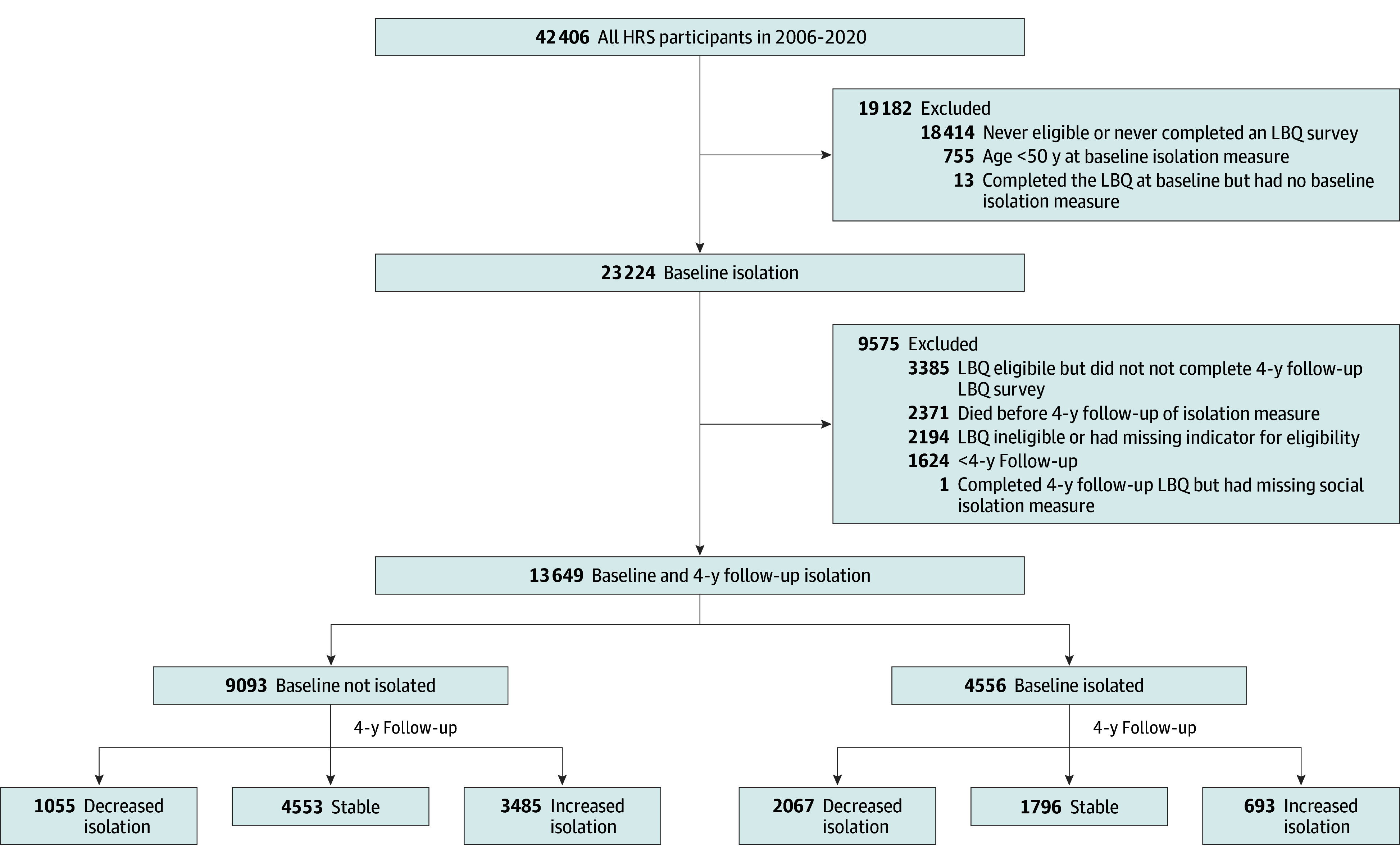
Flowchart of the Analysis Cohort Selection Among the 42 406 Health and Retirement Study (HRS) respondents in 2006 to 2020, we excluded 28 757 individuals. For disability, dementia, cardiovascular disease, and stroke outcomes, we further excluded individuals with no linked Medicare claims or with the outcome missing or the outcome before first measurement of social isolation. The flowcharts for the other 4 outcomes are listed in eFigure 1 in Supplement 1. LBQ indicates Leave-Behind Questionnaires.

The analysis cohort comprised 13 649 HRS respondents (mean [SD] age at baseline, 65.3 [9.5] years; 8011 female [58.7%]; 5638 male [41.3%]) from the 2006 to 2020 waves, aged 50 years or older at baseline, with nonmissing baseline SII measurements and nonmissing second SII measurements 4 years after baseline. Detailed sample exclusions are provided in Figure 1. Among these respondents, 9093 individuals (66.6%) were not socially isolated at baseline and 4556 (33.4%) were socially isolated. Of the baseline nonisolated respondents, 1055 (11.6%) experienced decreased isolation, 4553 (50.1%) remained stable, and 3485 (38.3%) experienced increased isolation at their second SII measurement. The SII changed by a mean (SD) of −1.0 (0) points for the decreased isolation, 0 (0) points for stable status, and 1.3 (0.62) points for the increased isolation groups over 4 years (eTable 2 in Supplement 1). Among the 4556 baseline isolated respondents, 2067 (45.4%) experienced decreased isolation, 1796 (39.4%) remained stable, and 693 (15.2%) experienced increased isolation at the second SII measurement. The SII changed by a mean (SD) of −1.37 (0.63) points for the decreased isolation, 0 (0) points for stable status, and 1.41 (0.60) points for the increased isolation groups (eTable 2 in Supplement 1). The flowchart for the 4 other outcomes is included in eFigure 1 in Supplement 1.

The demographic and clinical characteristics of the 3 isolation change groups are summarized in the Table. Compared with the decreased isolation group, the increased isolation group tended to be older, female, married, and non-Hispanic White and had lower levels of education. At baseline, they also tended to have higher total assets, poorer physical and cognition function, and a higher prevalence of comorbid conditions (eg, hypertension, CVD, and arthritis). Between baseline and the second SII measurement, they experienced higher incidences of stroke and psychiatric problems, increased levels of depression, and worsened physical and cognitive function declines. The demographic characteristics stratified by baseline social isolation status are summarized in eTable 3 in Supplement 1. There were no significant differences in follow-up periods among the 3 comparison groups for any outcome.

**Table.** Demographic and Clinical Characteristics of the HRS Analysis Cohort by Social Isolation Group From Baseline to Second Social Isolation Index Measurement

Characteristic	Participants, No. (%)			*P* value^a^
	Decreased isolation	Stable	Increased isolation	
Patients, No.	3122	6349	4178	NA
Sex				
Male	1432 (45.9)	2537 (40.0)	1669 (39.9)	<.001
Female	1690 (54.1)	3812 (60.0)	2509 (60.1)	
Cohort^b^				
AHEAD	98 (3.1)	236 (3.7)	241 (5.8)	<.001
CODA	264 (8.5)	597 (9.4)	553 (13.2)	
HRS	1023 (32.8)	2178 (34.3)	1436 (34.4)	
WB	474 (15.2)	1007 (15.9)	578 (13.8)	
BB	1263 (40.5)	2331 (36.7)	1370 (32.8)	
Race and ethnicity				
Hispanic	349 (11.2)	559 (8.8)	424 (10.1)	<.001
Non-Hispanic Black	526 (16.8)	903 (14.2)	645 (15.4)	
Non-Hispanic White	2153 (69.0)	4727 (74.5)	3003 (71.9)	
Other^c^	94 (3.0)	160 (2.5)	106 (2.5)	
Educational level, y				
<12	599 (19.2)	1092 (17.2)	853 (20.4)	<.001
12	1026 (32.9)	2131 (33.6)	1423 (34.1)	
>12	1497 (48.0)	3126 (49.2)	1902 (45.5)	
Baseline characteristics				
Age, mean (SD), y	64.37 (9.26)	64.99 (9.33)	66.56 (9.96)	<.001
Proxy vs self	3086 (98.8)	6266 (98.7)	4129 (98.8)	.75
Baseline isolation	2067 (66.2)	1796 (28.3)	693 (16.6)	<.001
Baseline year				
2006	1236 (39.6)	2498 (39.3)	1707 (40.9)	.008
2008	1032 (33.1)	2259 (35.6)	1489 (35.6)	
2010	432 (13.8)	796 (12.5)	470 (11.2)	
2012	341 (10.9)	655 (10.3)	403 (9.6)	
2014	81 (2.6)	141 (2.2)	109 (2.6)	
BMI, mean (SD)	28.79 (5.92)	28.67 (6.01)	28.55 (5.84)	.24
Hypertension	1623 (52.0)	3345 (52.7)	2290 (54.8)	.03
Diabetes	587 (18.8)	1120 (17.6)	785 (18.8)	.22
Lung disease	240 (7.7)	438 (6.9)	325 (7.8)	.17
Heart disease	575 (18.4)	1155 (18.2)	874 (20.9)	.001
Stroke	186 (6.0)	339 (5.3)	262 (6.3)	.12
Cancer	378 (12.1)	759 (12.0)	543 (13.0)	.26
Psychiatric problems	466 (14.9)	825 (13.0)	610 (14.6)	.01
Arthritis	1609 (51.5)	3453 (54.4)	2349 (56.2)	<.001
Total assets, quantile				
1st	713 (22.8)	1092 (17.2)	775 (18.5)	<.001
2nd	637 (20.4)	1235 (19.5)	910 (21.8)	
3rd	733 (23.5)	1577 (24.8)	1101 (26.4)	
4th	1039 (33.3)	2445 (38.5)	1392 (33.3)	
CES-D score, mean (SD)	1.44 (2.00)	1.25 (1.85)	1.43 (1.96)	<.001
Current smoker	437 (14.0)	851 (13.4)	582 (13.9)	.64
Married vs not married	1049 (33.6)	1917 (30.2)	1269 (30.4)	<.002
ADL score, mean (SD)	0.28 (0.84)	0.22 (0.74)	0.29 (0.84)	<.001
Cognition, mean (SD)^d^	15.60 (4.09)	16.03 (3.91)	15.34 (4.09)	<.001
Incidence disease from baseline to second social isolation measure				
Hypertension	263 (8.4)	506 (8.0)	339 (8.1)	.75
Diabetes	177 (5.7)	363 (5.7)	222 (5.3)	.66
Lung disease	93 (3.0)	199 (3.1)	138 (3.3)	.73
Heart disease	191 (6.1)	409 (6.4)	293 (7.0)	.28
Stroke	86 (2.8)	153 (2.4)	167 (4.0)	<.001
Cancer	130 (4.2)	250 (3.9)	179 (4.3)	.66
Psychiatric problem	92 (2.9)	187 (2.9)	178 (4.3)	<.001
Arthritis	248 (7.9)	471 (7.4)	334 (8.0)	.48
Change from baseline to second social isolation measure, mean (SD)				
CES-D score	−0.10 (1.62)	−0.02 (1.49)	0.07 (1.64)	<.001
ADL score	0.05 (0.71)	0.06 (0.66)	0.13 (0.79)	<.001
Cognition^d^	−0.43 (3.09)	−0.43 (2.95)	−0.58 (3.09)	.03

Abbreviations: ADL, activities of daily living; AHEAD, Assets and Health Dynamics Among the Oldest Old; BB, Baby Boomer; BMI, body mass index (calculated as weight in kilograms divided by height in meters squared); CES-D, Center for Epidemiological Studies–Depression; CODA, Children of the Depression; HRS, Health and Retirement Study; NA, not applicable; WB, War Baby.

^a^ *P* values are from the 2-sample *t* test or Wilcoxon rank sum test for comparing continuous covariates and the χ^2^ test for comparing categorical covariates.

^b^ The HRS included 5 birth cohorts: AHEAD, individuals born prior to 1924; CODA, born 1924-1930; original HRS, born 1931-1941; WB, born 1942-1947; and BB, born 1948-1959.

^c^ Other includes American Indian, Alaskan Native, Asian, Native Hawaiian and Pacific Islander, and other race or unknown.

^d^ Model-adjusted 27-point cognition score.

**Figure 2.**
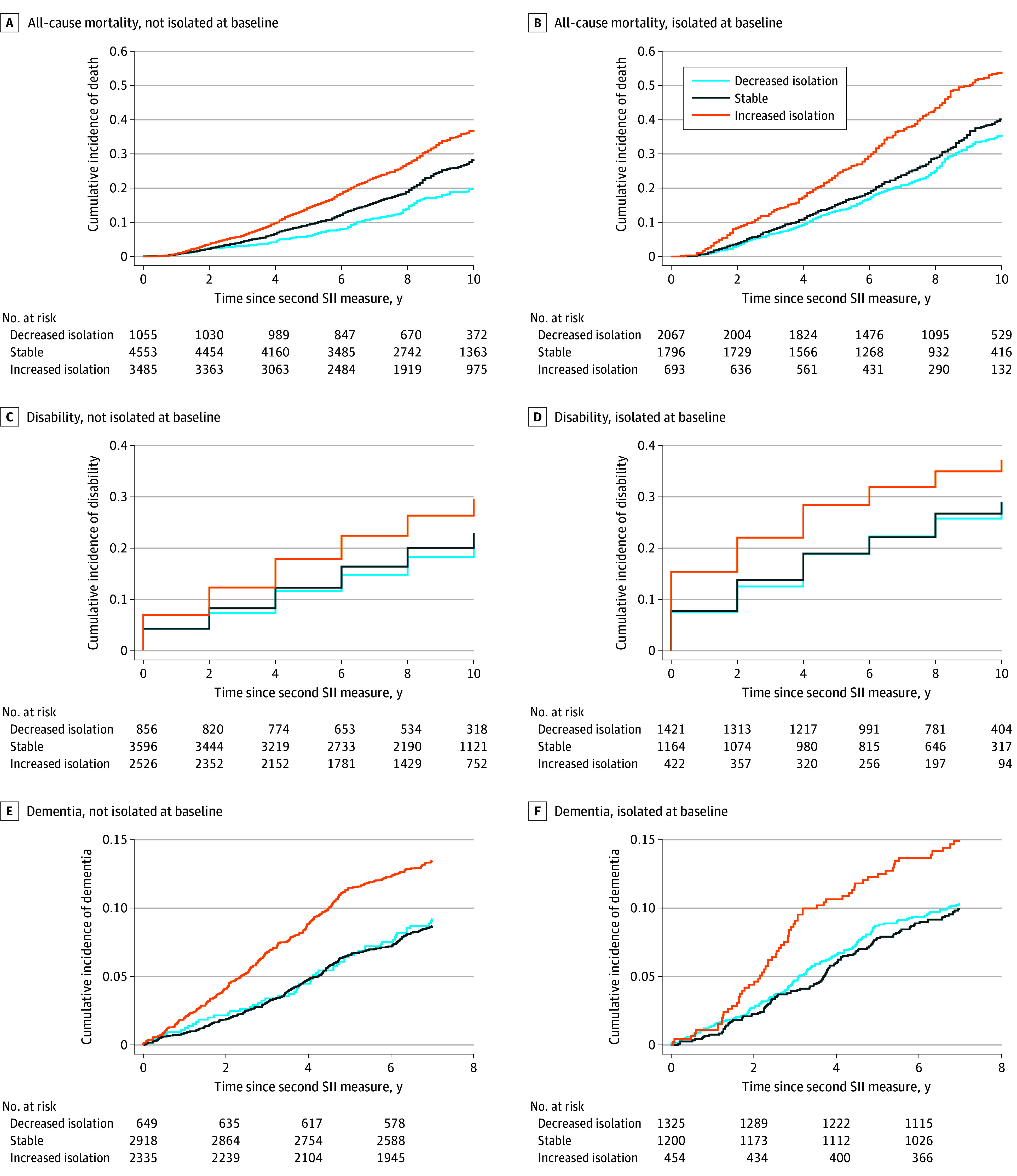
Cumulative Incidence Curves of Mortality, Disability, and Dementia for Social Isolation Change Groups, Stratified by Baseline Social Isolation Status Disability was measured as an activities of daily living score greater than 0, and dementia was defined as Alzheimer disease or Alzheimer disease–related dementia. The cumulative incidences for cardiovascular disease and stroke are listed in eFigure 3 in Supplement 1. SII indicates Social Isolation Index.

Cumulative incidence curves of mortality, disability, and dementia outcomes are shown in Figure 2. For mortality and disability, the increased isolation group exhibited the highest cumulative incidence, followed by the stable group, and then the decreased isolation group. These patterns were consistent for both baseline nonisolated and isolated respondents (Figure 2A-D). A higher incidence of dementia was observed for the increased isolation group, while the stable and decreased isolation groups had similar rates (Figure 2E-F). Minimal differences were observed for CVD and stroke among the 3 groups (eFigure 3 in Supplement 1).

**Figure 3.**
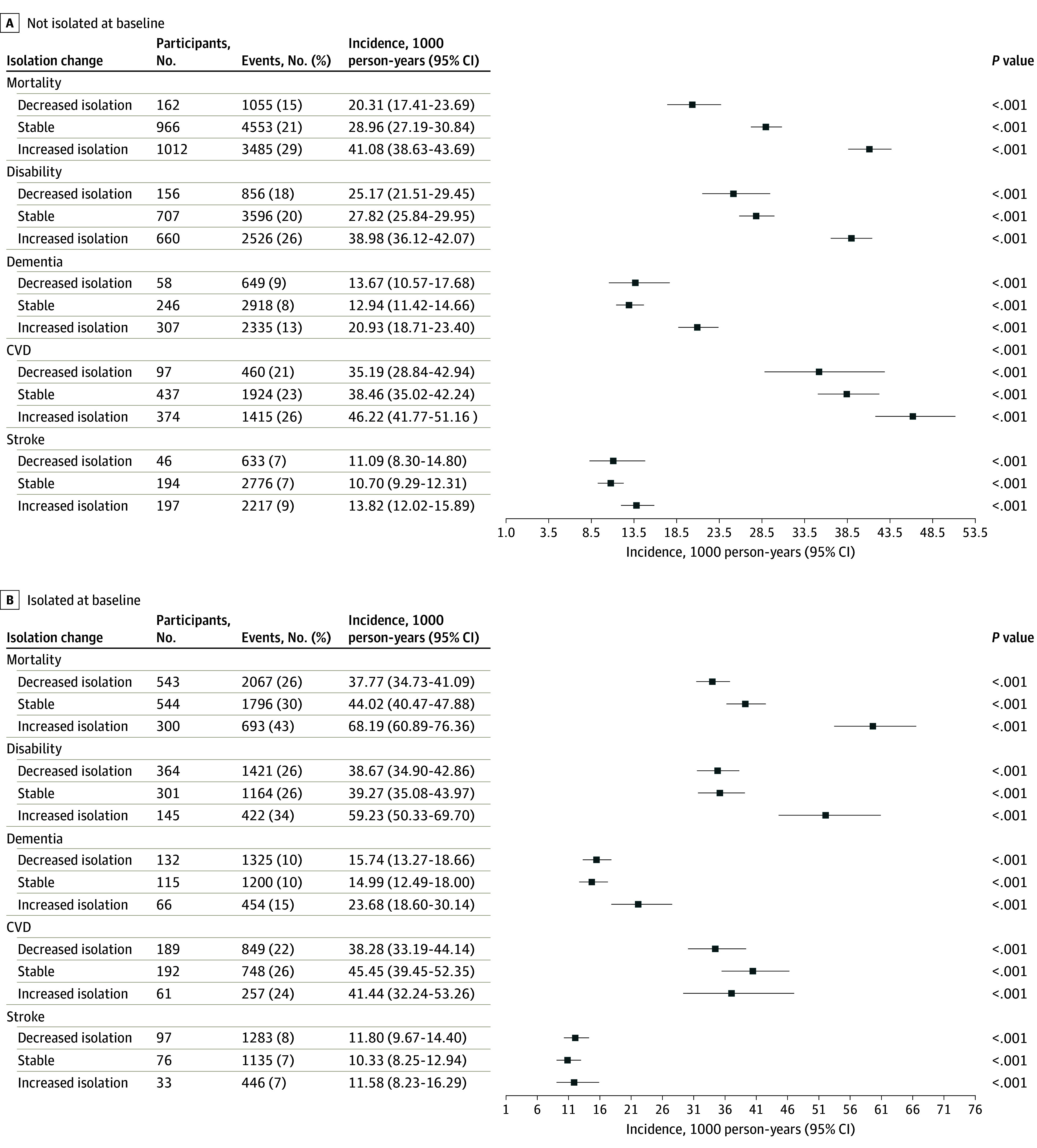
Incidence of Distal Outcomes for Social Isolation Change Groups, Stratified by Baseline Social Isolation Status Disability was measured as an activities of daily living score greater than 0; dementia included Alzheimer disease or Alzheimer disease–related dementia. CVD indicates cardiovascular disease; error bars indicate 95% CI.

Figure 3 presents the number of events per the number at risk and IRs of the studied outcomes for each isolation change group, stratified by isolation status at baseline. The increased isolation group exhibited higher incidences of mortality, disability, and dementia compared with the stable status and decreased isolation groups, regardless of baseline isolation status. For example, among the respondents who were not isolated at baseline, the estimated mortality IR was 20.31 (95% CI, 17.41-23.69) per 1000 person-years for the decreased isolation, 28.96 (95% CI, 27.19-30.84) per 1000 person-years for the stable status, and 41.08 (95% CI, 38.63-43.69) per 1000 person-years for the increased isolation groups. Among the participants who were isolated at baseline, the corresponding IRs were 37.77 (95% CI, 34.73-41.09) per 1000 person-years for decreased isolation, 44.02 (95% CI, 40.47-47.88) per 1000 person-years for stable status, and 68.19 (95% CI, 60.89-76.36) per 1000 person-years for increased isolation. Higher IRs were observed for CVD and stroke outcomes in the increased isolation group compared with the other 2 groups, but only among respondents who were not isolated at baseline.

**Figure 4.**
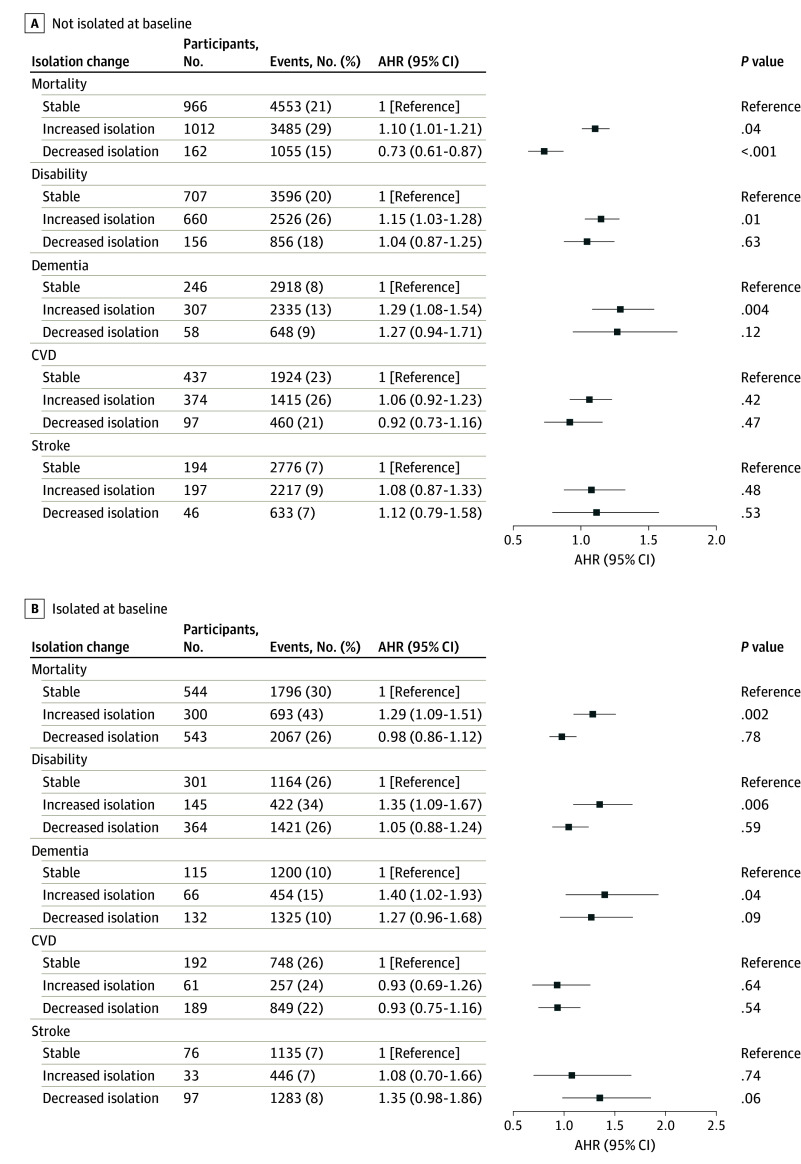
Adjusted Hazard Ratios (AHRs) of Changes in Isolation of Distal Outcomes, Stratified by Baseline Isolation Status The AHRs were weighted by probability for treatment weights. CVD indicates cardiovascular disease; error bars indicate 95% CI.

With the application of IPTW, adjusted covariates were well balanced among the 3 isolation change groups, with no significant standardized mean difference (eFigure 2 in Supplement 1). Figure 4 shows the IPTW weighted and covariates-adjusted hazard ratios (AHRs) of changes in isolation on 5 outcomes, stratified by baseline isolation status. Among respondents who were socially isolated at baseline, the increased social isolation group exhibited significantly higher hazards for mortality (AHR, 1.29; 95% CI, 1.09-1.51; *P* = .002), disability (AHR, 1.35; 95% CI, 1.09-1.67; *P* = .006), and dementia (AHR, 1.40; 95% CI, 1.02-1.93; *P* = .04) compared with the stable group. Similarly, among respondents who were not socially isolated at baseline, the increased isolation group showed significantly higher hazards of mortality (AHR, 1.10; 95% CI, 1.01-1.21; *P* = .04), disability (AHR, 1.15; 95% CI, 1.03-1.28; *P* = .01), and dementia (AHR, 1.29; 95% CI, 1.08-1.54; *P* = .004) compared with the stable group. Decreased isolation was associated with a lower risk of mortality (AHR, 0.73; 95% CI, 0.61-0.87; *P* < .001) solely among respondents not socially isolated at baseline, with no association observed for other analyzed outcomes. There was no association observed between changes in isolation and the risk of CVD or stroke outcomes. Hazard ratios of full covariates for mortality, disability, and dementia outcomes are summarized in eTable 4 in Supplement 1. Unadjusted HRs without IPTW are provided in eFigure 4 in Supplement 1.

In a sensitivity analysis, individuals transitioning from nonisolation to isolation showed significantly higher risks of mortality (AHR, 1.14; 95% CI, 1.04-1.26; *P* = .007), disability (AHR, 1.19; 95% CI, 1.05-1.34; *P* = .004), and dementia (AHR, 1.23; 95% CI, 1.03-1.48; *P* = .02) compared with the stable nonisolation group (eFigure 5 in Supplement 1). No significant difference was observed for any of the 5 outcomes between transitioning from isolation to nonisolation and the consistently isolated group. The conclusion remained consistent in additional sensitivity analyses by excluding individuals who died within 2 years after the second SII measure (eFigure 6 in Supplement 1) and by weighting using HRS sampling weights at baseline (eFigure 7 in Supplement 1).

## Discussion

In our study of a national cohort of US individuals aged 50 years or older, we found that changes in social isolation during 4 years had a long-term association with distal outcomes. In contrast to existing studies that often use cross-sectional measurements of social isolation, our study captures changes in isolation levels and categorizes these changes into groups: decreased isolation, stable isolation, and increased isolation groups. Increased isolation was consistently associated with increased risks of mortality, disability, and dementia, irrespective of the individual’s isolation status at baseline. These results suggest a need for interventions aimed at averting increases in isolation among older adults as a means to mitigate its adverse outcomes regarding mortality, as well as physical and cognitive function decline.

To our knowledge, our study represents the largest analysis to estimate the association between changes in social isolation and health outcomes. Our results align with the limited existing literature on this topic. For instance, a study analyzing 11 234 participants from the English Longitudinal Study of Aging concluded that increased social isolation predicted memory decline over 6 waves in women.^23^ Another US cohort–based analysis found that severe isolation over 8 years had the worst heath outcome.^22^

Increased social isolation is associated with worse health outcomes through several potential biological, behavioral, and psychological mechanisms. Cole et al^36^ reported that individuals with more social isolation had increased expression of genes related to proinflammatory cytokine signaling and prostaglandin synthesis. Increased expression of these genes can lead to glucocorticoid resistance,^37^ contributing to inflammation, oxidative stress, aging,^38^ chronic disease,^39^ hypertension, atherosclerosis,^37,40^ and mortality.^41,42,43^ Additionally, social isolation can activate the hypothalamic-pituitary-adrenal axis and the sympathetic nervous system, leading to behavioral alteration, such as physical inactivity, smoking, and disrupted sleep.^37^ Moreover, a bidirectional association between social isolation and dementia through neurogenesis related to α-amino-3-hydroxy-5-methyl-4-isoxazolepropionic acid receptor and brain-derived neurotrophic factor proteins^44^ has been reported.^45,46^ Behaviorally, increased social isolation may result in decreased engagement in social activities, reduced physical exercise, changes in dietary habits, and increased stress, depression, and anxiety, all of which can exacerbate its negative impact on health.

Conversely, our analysis revealed that a decrease in isolation was not associated with a lower risk of any studied outcomes except for mortality among respondents who initially were not isolated. The decrease in isolation did not show significance with any outcomes among individuals who were initially isolated. This finding suggests potential complexities in the association between changes in isolation and health outcomes. Factors such as baseline isolation status, duration of isolation, and magnitude of isolation decrease may influence the observed lack of associations. Methodological limitations in measuring isolation and health outcomes further underscore the need for additional research to better understand these dynamics.

### Strengths and Limitations

Our study has several unique strengths. First, the use of the HRS, with a large sample size and multiple variables collected longitudinally on respondents over 2 decades, enables powerful analysis of the long-term association between social isolation changes and distal outcomes among older adults. Second, the definitions of the social isolation change (from initial baseline SII measurement to the second SII measurement 4 years after baseline) and survival outcomes (from the second SII measurement to events) naturally establish the temporal sequence between exposure and outcome, ensuring the direction of the association. Third, through the application of IPTW, we ensure that the 3 change groups are balanced with respect to the analyzed covariates at the onset of the time-to-event outcomes. Fourth, the analysis was stratified by the social isolation status at baseline, allowing for exploration of heterogeneity associations based on the initial status of social isolation.

Our study also has limitations. Despite rigorous analysis, the HRS is a cohort study, which cannot establish causality. Additionally, the change in social isolation is limited to a 4-year span. Furthermore, the SII is defined based on only 5 questions, which may result in a ceiling effect due to its narrow range. The outcome events may be underestimated as Medicare may not fully capture CVD, stroke, and dementia diagnoses. Future studies would benefit from using finer measurements to capture a more comprehensive understanding of the impact of interventions targeting changes in social isolation. Interventions targeting social isolation in older adults are inherently complex and may have limitations in their efficacy or scope. For example, life events, such as the death of a spouse, can lead to increased social isolation and cannot be prevented. Our analysis indicates that interventions, such as increased community outreach and psychological therapies, aimed at avoiding increased social isolation in the presence of such life events may mitigate the risk of adverse outcomes. Addressing the inevitability of certain life events and incorporating this reality into intervention strategies is crucial for reducing social isolation and improving health outcomes.

## Conclusions

This cohort study found an association between increased isolation and greater risks of mortality, disability, and dementia in older adults. Conversely, decreased isolation was associated with a reduced risk of mortality only among individuals who were not socially isolated at baseline. These results underscore the importance of interventions targeting the prevention of increased isolation among older adults to mitigate its adverse effects on mortality, as well as physical and cognitive function.

## Notes

Supplement 1. **eFigure 1.** Flow chart of the study design **eFigure 2.** The standardized mean difference for covariates **eFigure 3.** The cumulative incidence curves of CVD and stroke for social isolation change from baseline to second isolation measure and the social isolation status **eFigure 4.** Unadjusted hazard ratios of changes in isolation on distal outcomes stratified by the social isolation status **eFigure 5.** Sensitivity analysis 1: adjusted hazard ratios of changes in binary isolation group on distal outcomes stratified by the social isolation status **eFigure 6.** Sensitivity analysis 2: adjusted hazard ratios of changes in isolation group on distal outcomes among individuals who did not die within 2 years post second SII measurement **eFigure 7.** Sensitivity analysis 3: adjusted hazard ratios of changes in isolation group on distal outcomes stratified by the social isolation status incorporating HRS sampling weights **eTable 1.** *ICD-10* diagnosis codes from Medicare chronic conditions warehouse **eTable 2.** Social isolation index change from baseline to second social isolation measurement stratified by isolation status **eTable 3.** Demographic and clinical characteristics of the HRS analysis cohort by social isolation group from baseline to second social isolation measure **eTable 4.** The hazard ratios of full covariates on distal outcomes stratified by the social isolation status

Supplement 2.
Data Sharing Statement
