# Supplementary material for: Social Isolation Changes and Long-Term Outcomes Among Older Adults
Source: JAMA Netw Open. 2024 Jul 24;7(7):e2424519. doi: 10.1001/jamanetworkopen.2024.24519 (PMC11270134; doi:10.1001/jamanetworkopen.2024.24519)
Supplement: Supplement 1. — eFigure 1. Flow chart of the study design eFigure 2. The standardized mean difference for covariates eFigure 3. The cumulative incidence curves of CVD and stroke for social isolation change from baseline to second isolation measure and the social isolation status eFigure 4. Unadjusted hazard ratios of changes in isolation on distal outcomes stratified by the social isolation status eFigure 5. Sensitivity analysis 1: adjusted hazard ratios of changes in binary isolation group on distal outcomes stratified by the social isolation status eFigure 6. Sensitivity analysis 2: adjusted hazard ratios of changes in isolation group on distal outcomes among individuals who did not die within 2 years post second SII measurement eFigure 7. Sensitivity analysis 3: adjusted hazard ratios of changes in isolation group on distal outcomes stratified by the social isolation status incorporating HRS sampling weights eTable 1. ICD-10 diagnosis codes from Medicare chronic conditions warehouse eTable 2. Social isolation index change from baseline to second social isolation measurement stratified by isolation status eTable 3. Demographic and clinical characteristics of the HRS analysis cohort by social isolation group from baseline to second social isolation measure eTable 4. The hazard ratios of full covariates on distal outcomes stratified by the social isolation status [file jamanetwopen-e2424519-s001.pdf]

Supplemental Online Content

Lyu C, Siu K, Xu I, Osman I, Zhong J. Social isolation changes and long-term outcomes among older adults. *JAMA Netw Open*. 2024;7(7):e2424519. doi:10.1001/jamanetworkopen.2024.24519

eFigure 1. Flow chart of the study design

eFigure 2. The standardized mean difference for covariates

eFigure 3. The cumulative incidence curves of CVD and stroke for social isolation change from baseline to second social isolation measure and the social isolation status

eFigure 4. Unadjusted hazard ratios of changes in isolation on distal outcomes stratified by the social isolation status

eFigure 5. Sensitivity analysis 1: adjusted hazard ratios of changes in binary isolation group on distal outcomes stratified by the social isolation status

eFigure 6. Sensitivity analysis 2: Adjusted hazard ratios of changes in isolation group on distal outcomes among individuals who did not die within 2 years post second SII measurement

eFigure 7. Sensitivity analysis 3: Adjusted hazard ratios of changes in isolation group on distal outcomes stratified by the social isolation status incorporating HRS sampling weights

eTable 1. ICD-10 Diagnosis codes from Medicare Chronic Conditions Warehouse

eTable 2. Social Isolation Index change from baseline to second social isolation measurement stratified by isolation status

eTable 3. Demographic and clinical characteristics of the HRS analysis cohort by social isolation group from baseline to second social isolation measure

eTable 4. The hazard ratios of full covariates on distal outcomes stratified by the social isolation status

© 2024 Lyu C et al. *JAMA Network Open.*

This supplemental material has been provided by the authors to give readers additional information about their work.

© 2024 Lyu C et al. *JAMA Network Open.*

Online-Only Supplementary Material

Efigure 1: Flow chart of the study design.

The flow charts are listed for A) disability (ADL>0), B) Dementia (AD or ADRD), C) CVD (acute myocardial infarction, atrial fibrillation, congestive heart failure, ischemic heart disease or stroke) and D) stroke. In addition to the exclusion criteria in **Figure 1**, we further excluded the individuals with no linked Medicare claims or with the outcome missing or the outcome before first measurement of social isolation.

© 2024 Lyu C et al. *JAMA Network Open.*

A)

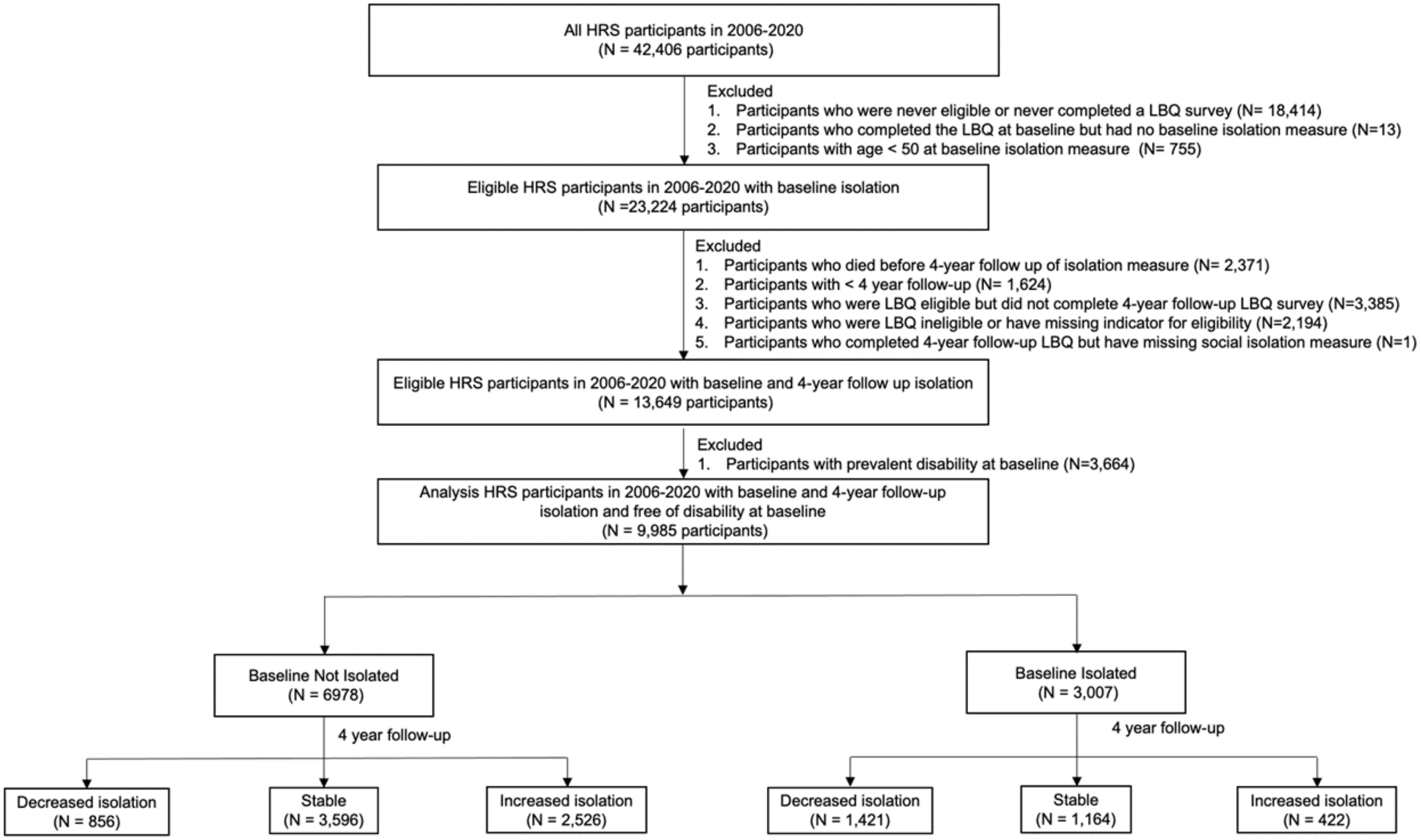

© 2024 Lyu C et al. *JAMA Network Open.*

B)

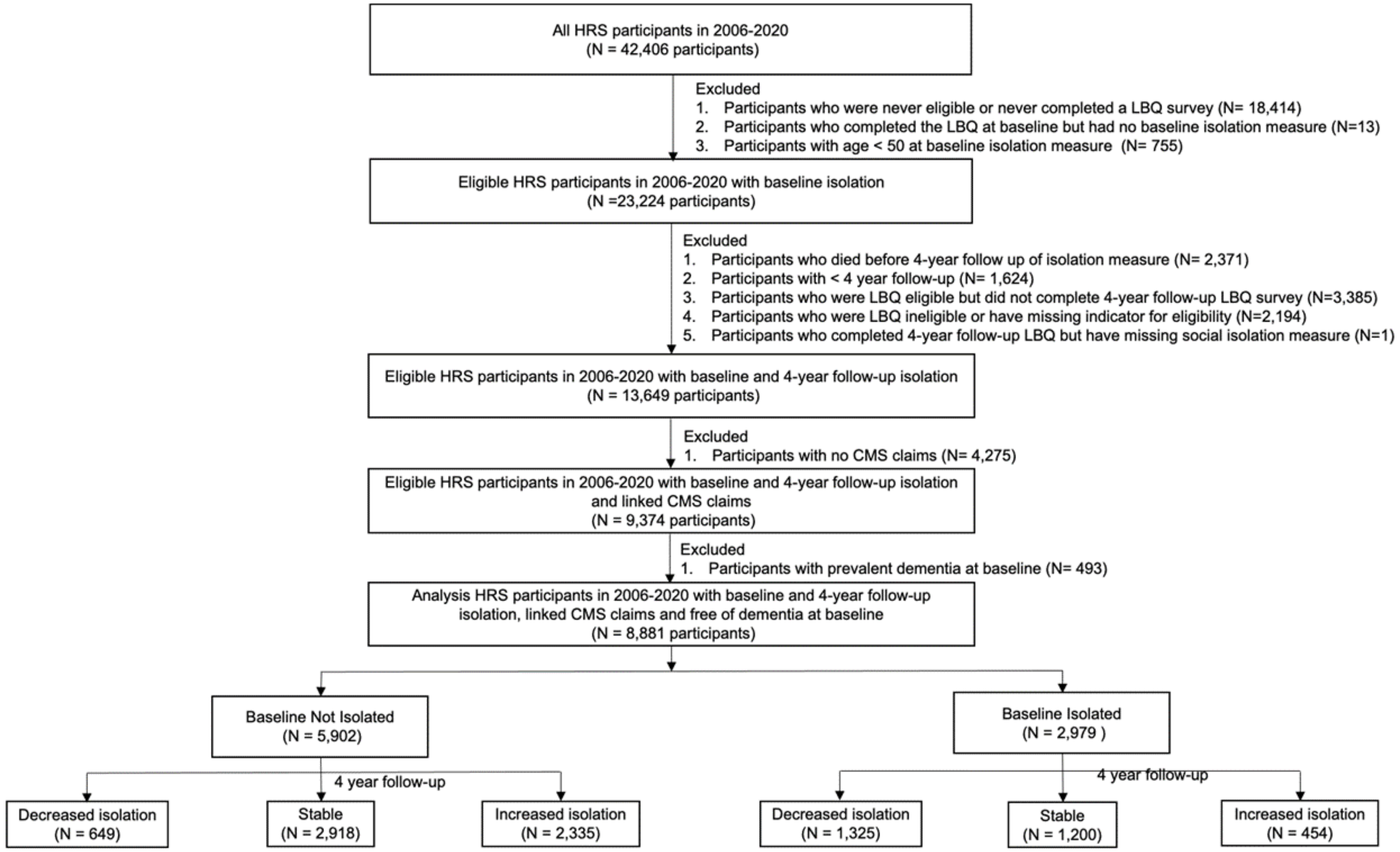

© 2024 Lyu C et al. *JAMA Network Open.*

C)

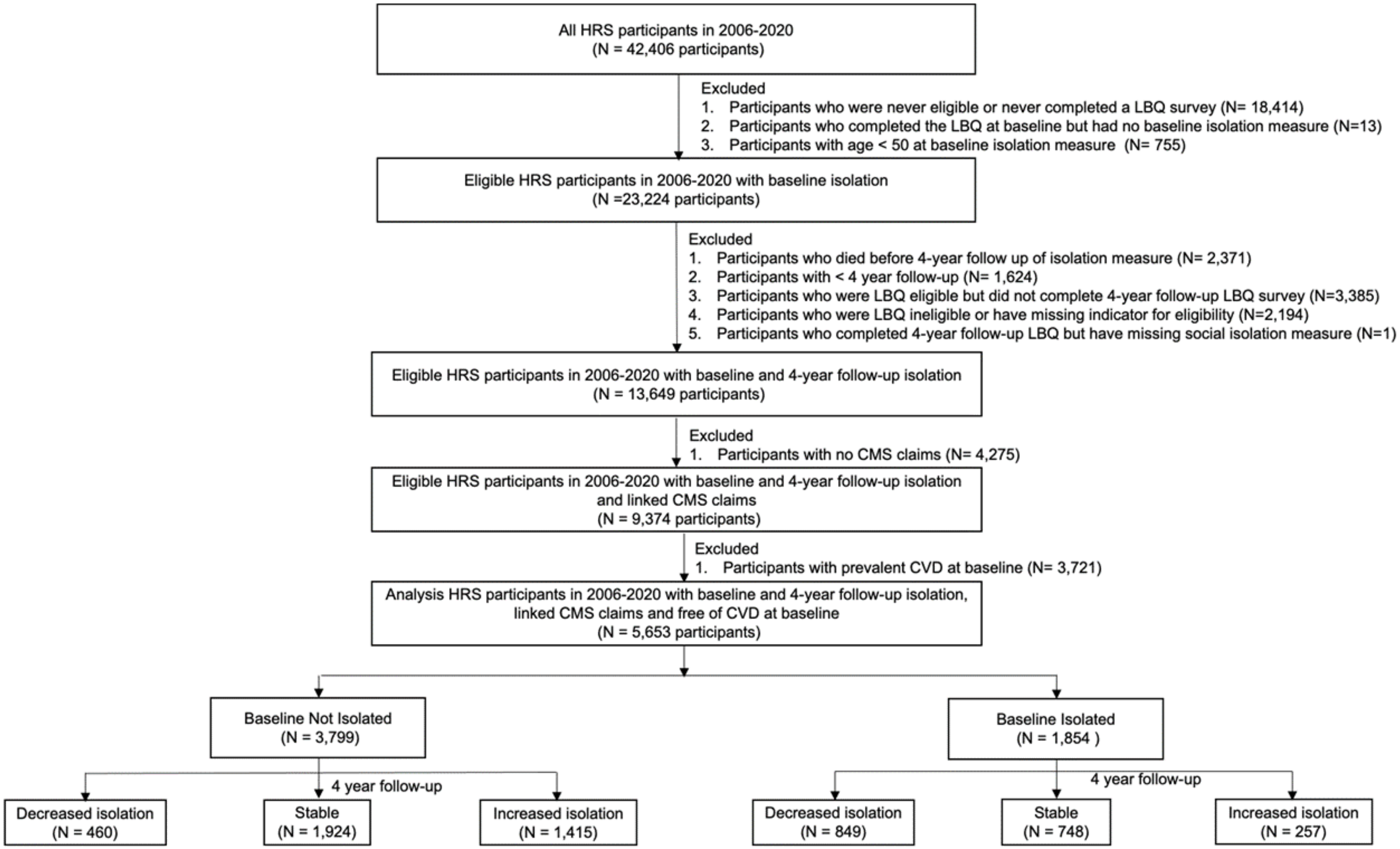

© 2024 Lyu C et al. *JAMA Network Open.*

D)

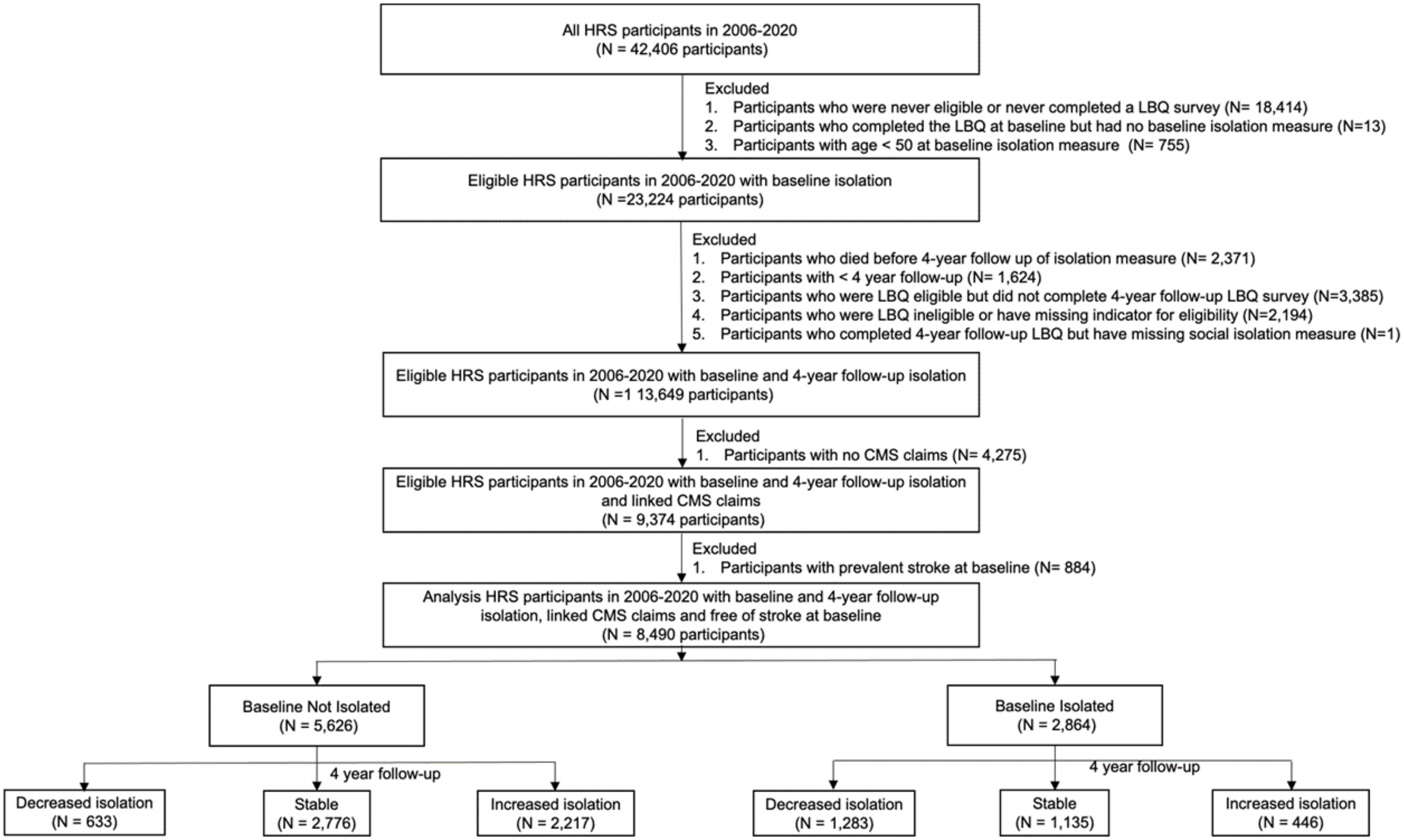

© 2024 Lyu C et al. *JAMA Network Open.*

Efigure 2: The standardized mean difference for covariates

The standardized mean difference (SMD) was calculated for each covariate before and after inverse probability of treatment weighting. The solid line refers to weighted SMD and the dash line represents the unadjusted one.

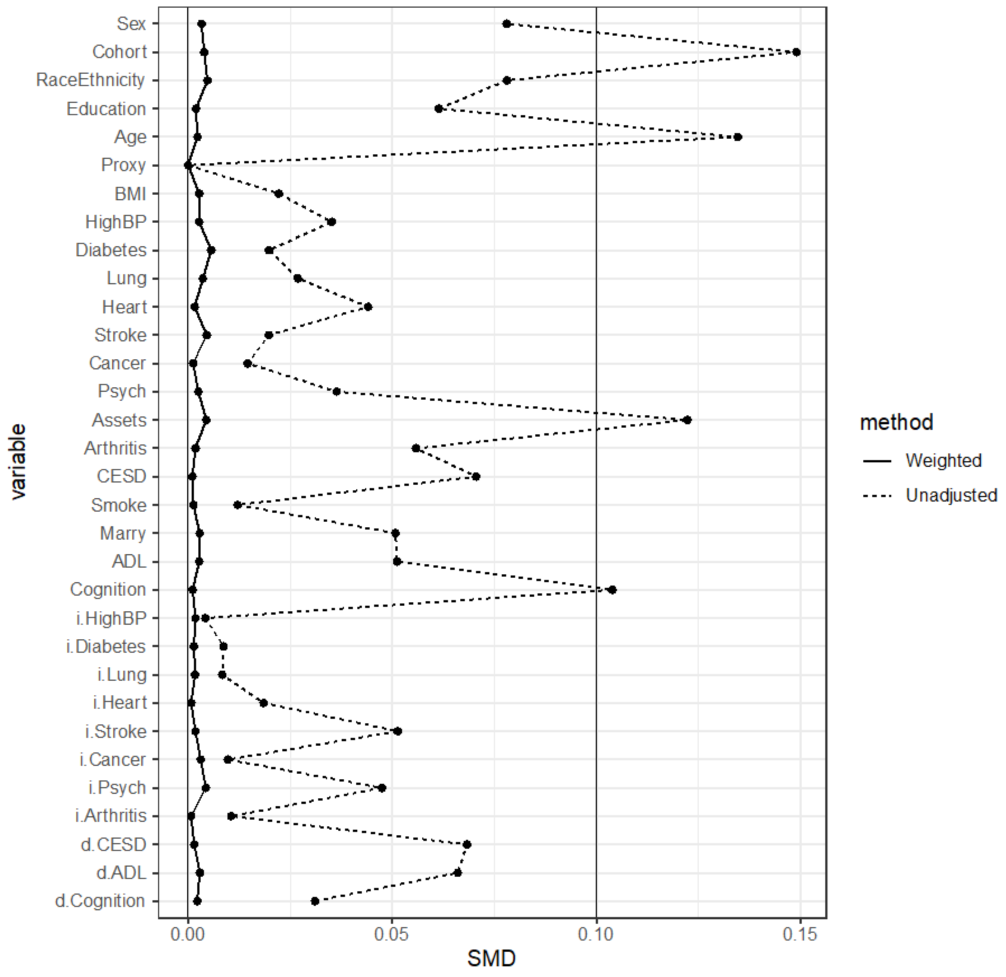

© 2024 Lyu C et al. *JAMA Network Open.*

Efigure 3: The cumulative incidence curves of CVD and stroke for social isolation change from baseline to second social isolation measure and the social isolation status.

The outcomes include A-B) CVD; and C-D) stroke. The figures were stratified by the social isolation status: A, C) baseline non-isolation and B, D) baseline isolation. The x-axis of each figure is the year since second social isolation measure. The year since second social isolation measure is the 4 year later since the first measurement of social isolation. The curves are colored by the three isolation change groups from baseline to second social isolation measure (blue: decreased isolation, purple: stable and orange: increased isolation). The number of participants at risk at each time point were listed in the table below each figure.

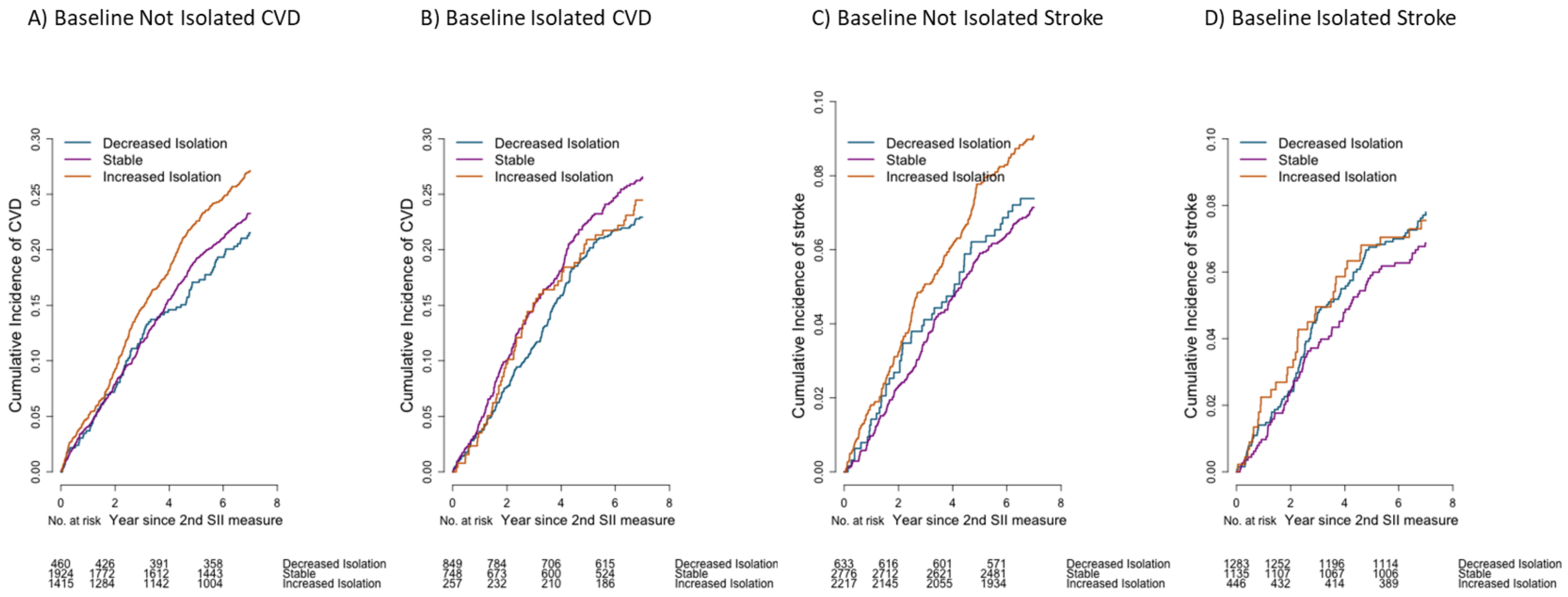

© 2024 Lyu C et al. *JAMA Network Open.*

Efigure 4: Unadjusted hazard ratios of changes in isolation on distal outcomes stratified by the social isolation status

The hazard ratios (95% confident interval) between isolation change group are estimated for each outcome with stable social isolation as the reference group with no covariate adjustment nor IPTW use. The p-value column presents the p-values testing the corresponding HR of diabetes to the null hypothesis (HR=1).

© 2024 Lyu C et al. *JAMA Network Open.*

A) Baseline Not Isolated

Outcome / Isolation change	Events/N(%)	aHR 95% CI	P value
**Mortality**			
Stable	966/4553(21)	1.00 (Ref)	Ref
Increased Isolation	1012/3485(29)	1.41(1.29-1.54)	<0.001
Decreased Isolation	162/1055(15)	0.68(0.58-0.81)	<0.001
**Disability**			
Stable	707/3596(20)	1.00 (Ref)	Ref
Increased Isolation	660/2526(26)	1.37(1.24-1.53)	<0.001
Decreased Isolation	156/856(18)	0.9(0.76-1.07)	0.25
**Dementia**			
Stable	246/2918(8)	1.00 (Ref)	Ref
Increased Isolation	307/2335(13)	1.61(1.36-1.91)	<0.001
Decreased Isolation	58/649(9)	1.07(0.81-1.43)	0.62
**CVD**			
Stable	437/1924(23)	1.00 (Ref)	Ref
Increased Isolation	374/1415(26)	1.19(1.04-1.37)	0.01
Decreased Isolation	97/460(21)	0.91(0.73-1.14)	0.41
**Stroke**			
Stable	194/2776(7)	1.00 (Ref)	Ref
Increased Isolation	197/2217(9)	1.29(1.05-1.57)	0.01
Decreased Isolation	46/633(7)	1.05(0.76-1.45)	0.77

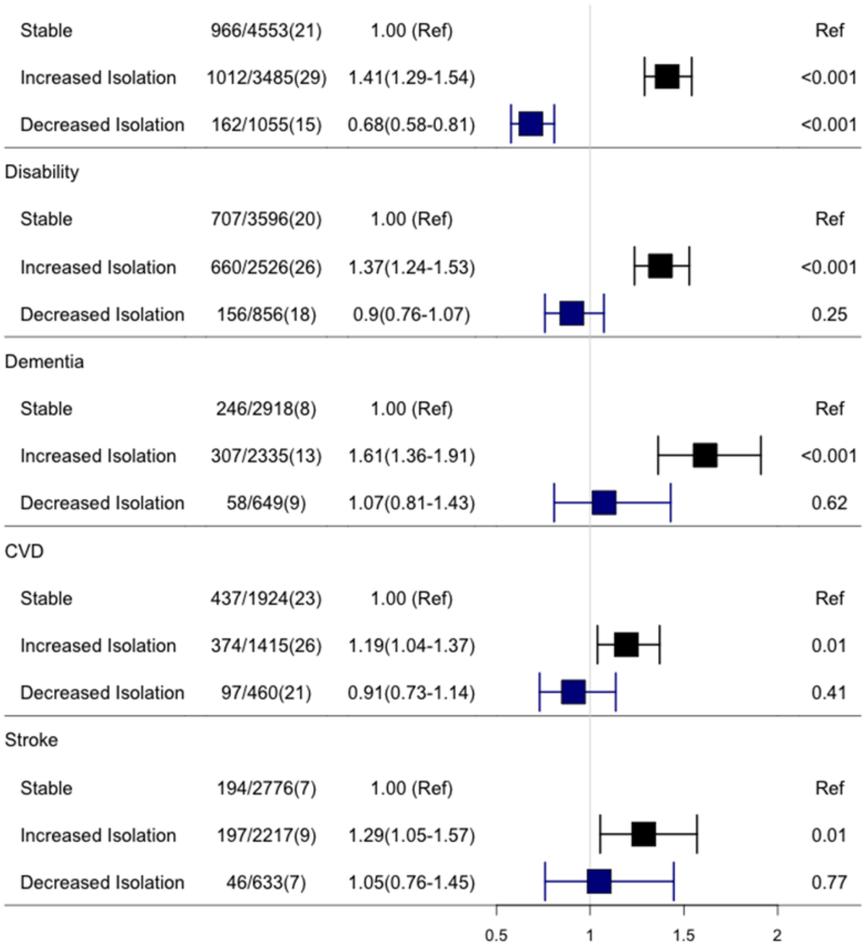

B) Baseline Isolated

Outcome / Isolation change	Events/N(%)	HR 95% CI	P value
**Mortality**			
Stable	544/1796(30)	1.00 (Ref)	Ref
Increased Isolation	300/693(43)	1.61(1.39-1.86)	<0.001
Decreased Isolation	543/2067(26)	0.88(0.78-1)	0.04
**Disability**			
Stable	301/1164(26)	1.00 (Ref)	Ref
Increased Isolation	145/422(34)	1.44(1.17-1.77)	0.001
Decreased Isolation	364/1421(26)	0.98(0.84-1.14)	0.80
**Dementia**			
Stable	115/1200(10)	1.00 (Ref)	Ref
Increased Isolation	66/454(15)	1.6(1.18-2.17)	0.002
Decreased Isolation	132/1325(10)	1.04(0.81-1.33)	0.78
**CVD**			
Stable	192/748(26)	1.00 (Ref)	Ref
Increased Isolation	61/257(24)	0.91(0.68-1.22)	0.55
Decreased Isolation	189/849(22)	0.85(0.69-1.04)	0.11
**Stroke**			
Stable	76/1135(7)	1.00 (Ref)	Ref
Increased Isolation	33/446(7)	1.14(0.76-1.72)	0.53
Decreased Isolation	97/1283(8)	1.13(0.83-1.53)	0.44

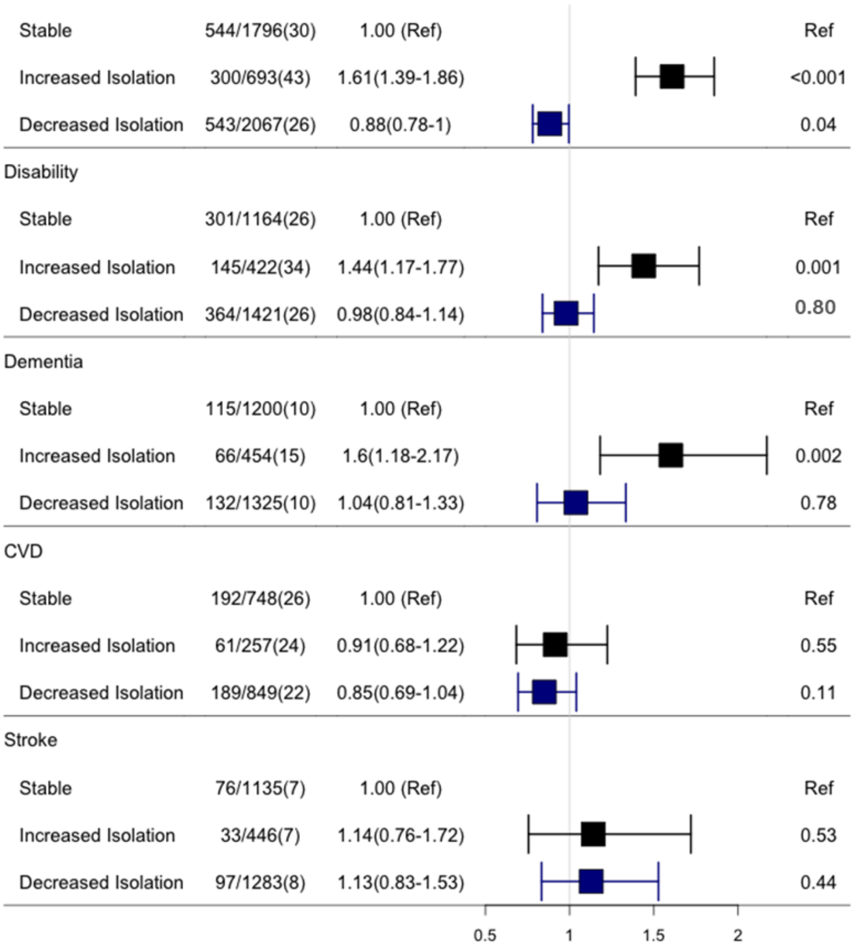

© 2024 Lyu C et al. *JAMA Network Open.*

Efigure 5: Sensitivity analysis 1: adjusted hazard ratios of changes in binary isolation group on distal outcomes stratified by the social isolation status

The hazard ratios (95% confident interval) between binary isolation change group are estimated for each outcome with stable social isolation as the reference group with covariate adjustment and IPTW use. The p-value column presents the p-values testing the corresponding HR of diabetes to the null hypothesis (HR=1).

© 2024 Lyu C et al. *JAMA Network Open.*

A) Baseline Not Isolated

Outcome / Isolation change	Events/N(%)	HR 95% CI	P value
**Mortality**			
Non Isolation-Non Isolation	966/4553(21)	1.00 (Ref)	Ref
Non Isolation-Isolation	1012/3485(29)	1.14(1.04-1.26)	0.007
**Disability**			
Non Isolation-Non Isolation	707/3596(20)	1.00 (Ref)	Ref
Non Isolation-Isolation	660/2526(26)	1.19(1.05-1.34)	0.004
**Dementia**			
Non Isolation-Non Isolation	246/2918(8)	1.00 (Ref)	Ref
Non Isolation-Isolation	307/2335(13)	1.23(1.03-1.48)	0.02
**CVD**			
Non Isolation-Non Isolation	437/1924(23)	1.00 (Ref)	Ref
Non Isolation-Isolation	374/1415(26)	1.11(0.95-1.3)	0.20
**Stroke**			
Non Isolation-Non Isolation	194/2776(7)	1.00 (Ref)	Ref
Non Isolation-Isolation	197/2217(9)	0.84(0.66-1.06)	0.14

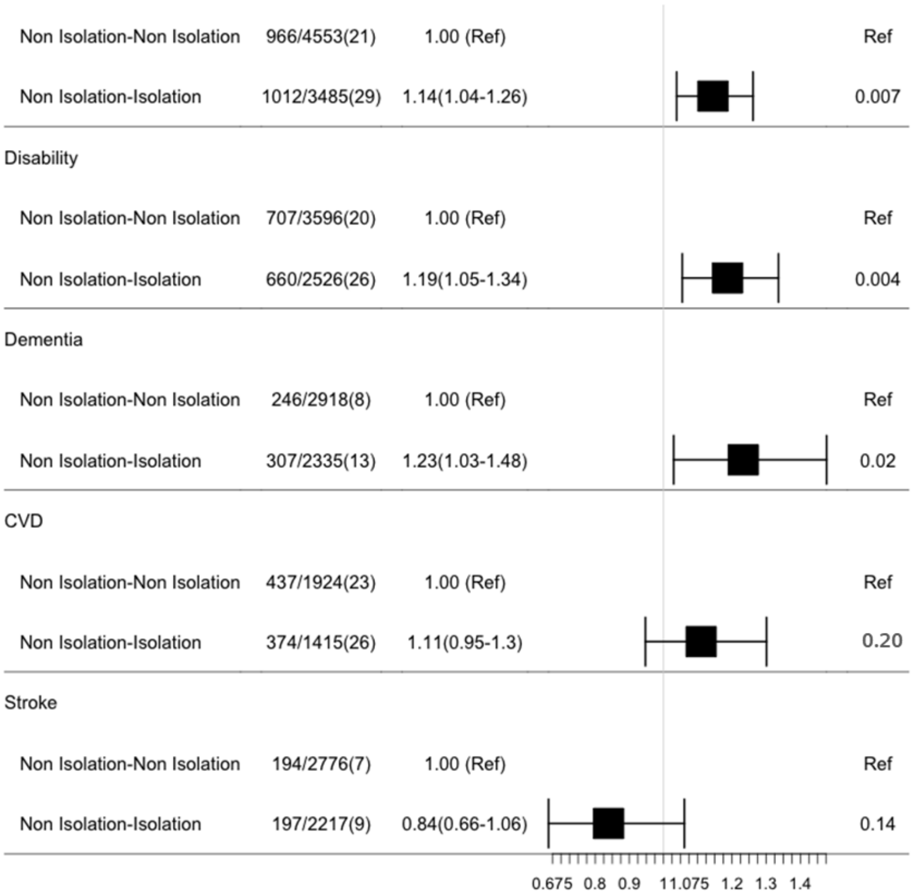

B) Baseline Isolated

Outcome / Isolation change	Events/N(%)	HR 95% CI	P value
**Mortality**			
Isolation-Isolation	544/1796(30)	1.00 (Ref)	Ref
Isolation-Non Isolation	543/2067(26)	0.9(0.79-1.03)	0.12
**Disability**			
Isolation-Isolation	301/1164(26)	1.00 (Ref)	Ref
Isolation-Non Isolation	364/1421(26)	0.97(0.82-1.14)	0.69
**Dementia**			
Isolation-Isolation	115/1200(10)	1.00 (Ref)	Ref
Isolation-Non Isolation	132/1325(10)	1.06(0.81-1.39)	0.65
**CVD**			
Isolation-Isolation	192/748(26)	1.00 (Ref)	Ref
Isolation-Non Isolation	189/849(22)	0.93(0.75-1.15)	0.49
**Stroke**			
Isolation-Isolation	76/1135(7)	1.00 (Ref)	Ref
Isolation-Non Isolation	97/1283(8)	1.33(0.98-1.8)	0.07

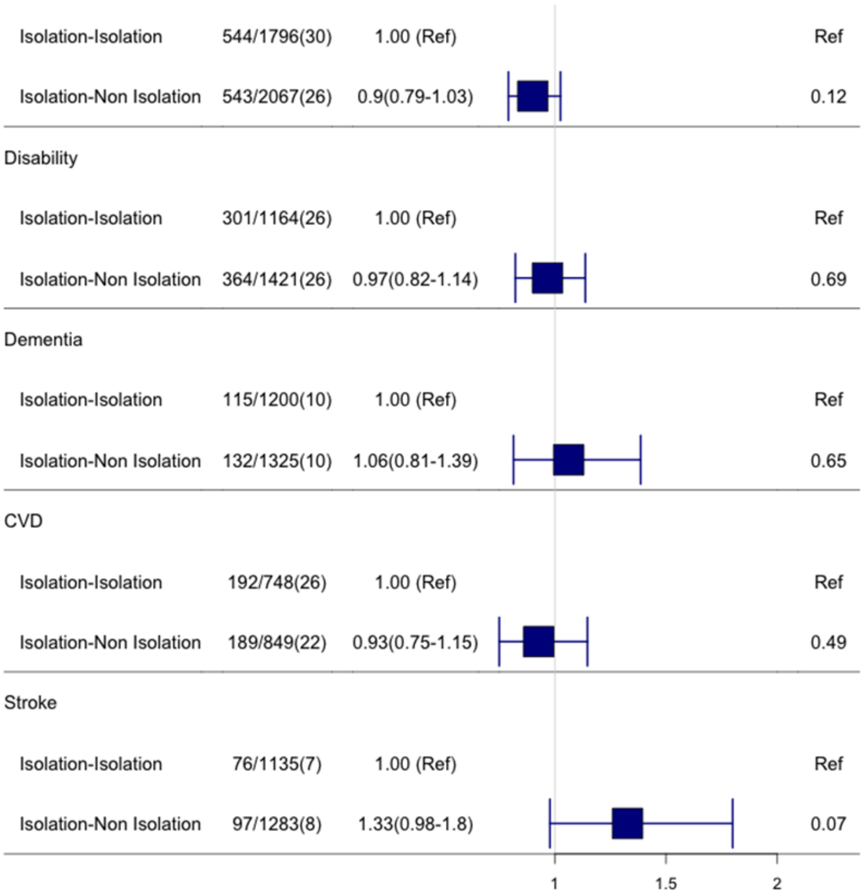

© 2024 Lyu C et al. *JAMA Network Open.*

Efigure 6: Sensitivity analysis 2: Adjusted hazard ratios of changes in isolation group on distal outcomes among individuals who did not die within 2 years post second SII measurement

The hazard ratios (95% confident interval) between isolation change group are estimated for each outcome with stable social isolation as the reference group with covariate adjustment and IPTW use. The p-value column presents the p-values testing the corresponding HR of diabetes to the null hypothesis (HR=1).

© 2024 Lyu C et al. *JAMA Network Open.*

A) Baseline Not Isolated

Outcome / Isolation change	Events/N(%)	aHR 95% CI	P value
**Mortality**			
Stable	854/4397(19)	1.00 (Ref)	Ref
Increased Isolation	870/3310(26)	1.1(1-1.21)	0.06
Decreased Isolation	134/1012(13)	0.68(0.56-0.82)	<0.001
**Disability**			
Stable	693/3499(20)	1.00 (Ref)	Ref
Increased Isolation	630/2426(26)	1.15(1.03-1.29)	0.01
Decreased Isolation	152/827(18)	1.06(0.88-1.26)	0.55
**Dementia**			
Stable	240/2814(9)	1.00 (Ref)	Ref
Increased Isolation	292/2218(13)	1.3(1.08-1.55)	0.004
Decreased Isolation	55/626(9)	1.24(0.91-1.69)	0.17
**CVD**			
Stable	427/1874(23)	1.00 (Ref)	Ref
Increased Isolation	367/1366(27)	1.1(0.95-1.27)	0.21
Decreased Isolation	94/450(21)	0.9(0.71-1.14)	0.39
**Stroke**			
Stable	187/2689(7)	1.00 (Ref)	Ref
Increased Isolation	191/2115(9)	1.12(0.91-1.38)	0.29
Decreased Isolation	43/613(7)	1.07(0.75-1.52)	0.71

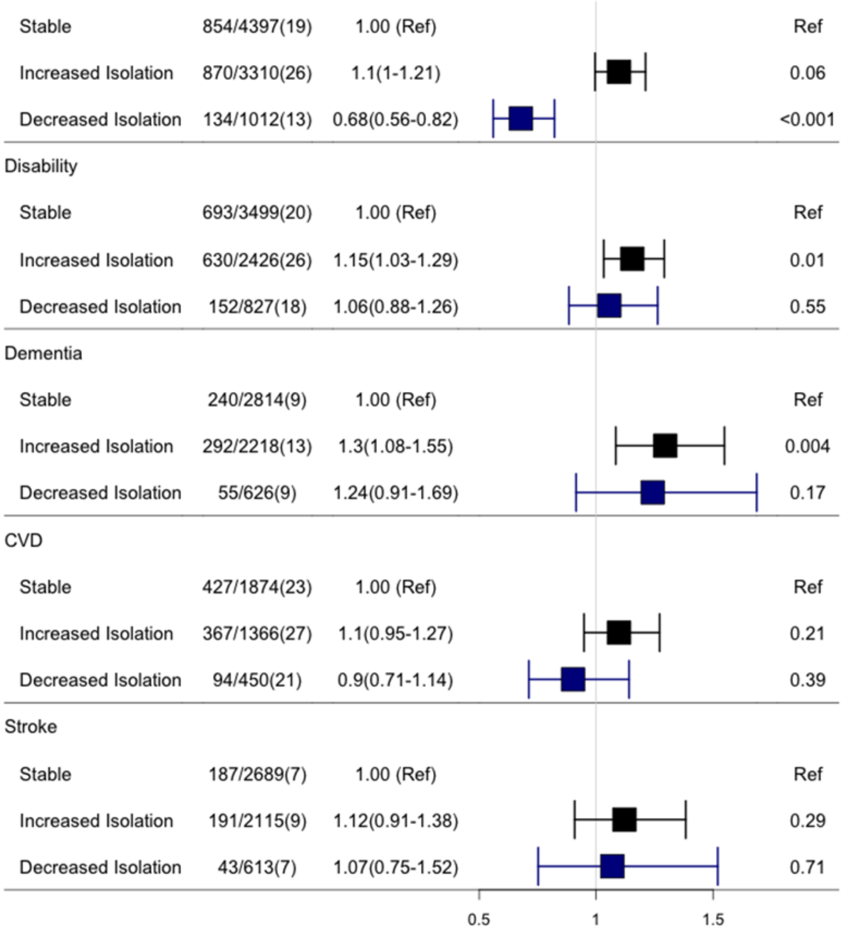

B) Baseline Isolated

Outcome / Isolation change	Events/N(%)	aHR 95% CI	P value
**Mortality**			
Stable	456/1694(27)	1.00 (Ref)	Ref
Increased Isolation	238/627(38)	1.26(1.06-1.5)	0.01
Decreased Isolation	466/1969(24)	0.99(0.85-1.14)	0.85
**Disability**			
Stable	285/1115(26)	1.00 (Ref)	Ref
Increased Isolation	132/392(34)	1.37(1.1-1.7)	0.005
Decreased Isolation	346/1372(25)	1.06(0.89-1.25)	0.54
**Dementia**			
Stable	113/1134(10)	1.00 (Ref)	Ref
Increased Isolation	64/412(16)	1.47(1.06-2.03)	0.02
Decreased Isolation	124/1262(10)	1.23(0.93-1.63)	0.15
**CVD**			
Stable	184/723(25)	1.00 (Ref)	Ref
Increased Isolation	57/244(23)	0.93(0.68-1.27)	0.65
Decreased Isolation	184/826(22)	0.93(0.75-1.17)	0.54
**Stroke**			
Stable	70/1071(7)	1.00 (Ref)	Ref
Increased Isolation	31/404(8)	1.2(0.77-1.85)	0.43
Decreased Isolation	92/1220(8)	1.42(1.02-1.96)	0.04

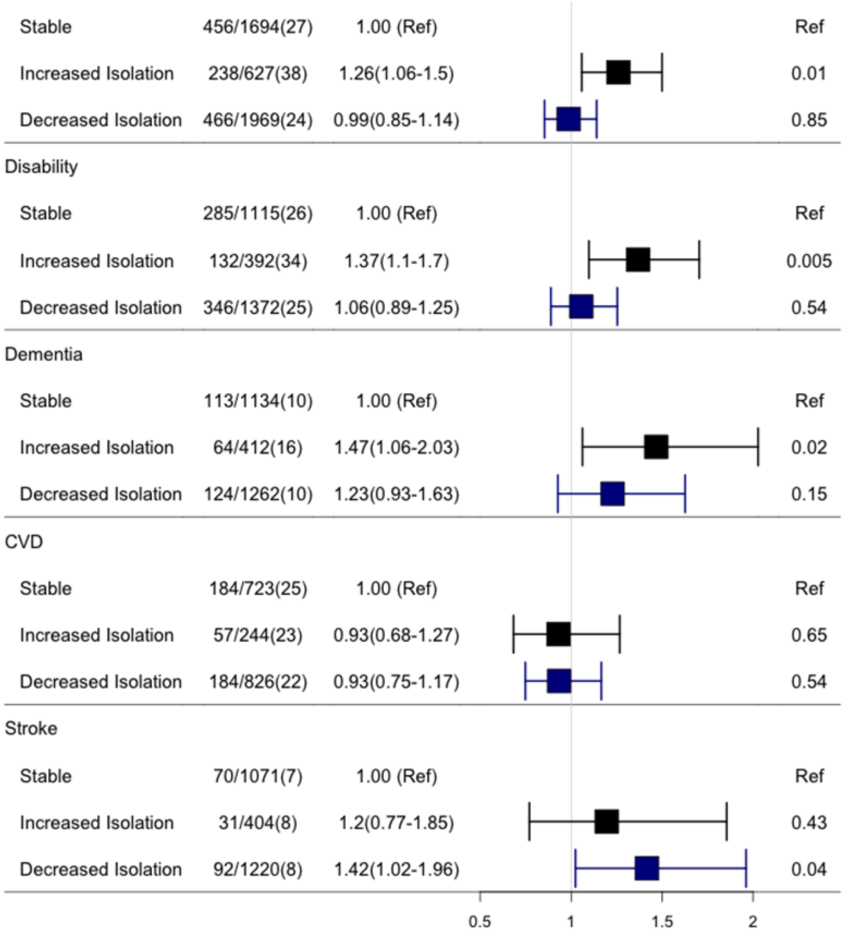

© 2024 Lyu C et al. *JAMA Network Open.*

Efigure 7: Sensitivity analysis 3: Adjusted hazard ratios of changes in isolation group on distal outcomes stratified by the social isolation status incorporating HRS sampling weights

The hazard ratios (95% confident interval) between isolation change group are estimated for each outcome with stable social isolation as the reference group with covariate adjustment, IPTW use and HRS sampling weights. The p-value column presents the p-values testing the corresponding HR of diabetes to the null hypothesis (HR=1).

© 2024 Lyu C et al. *JAMA Network Open.*

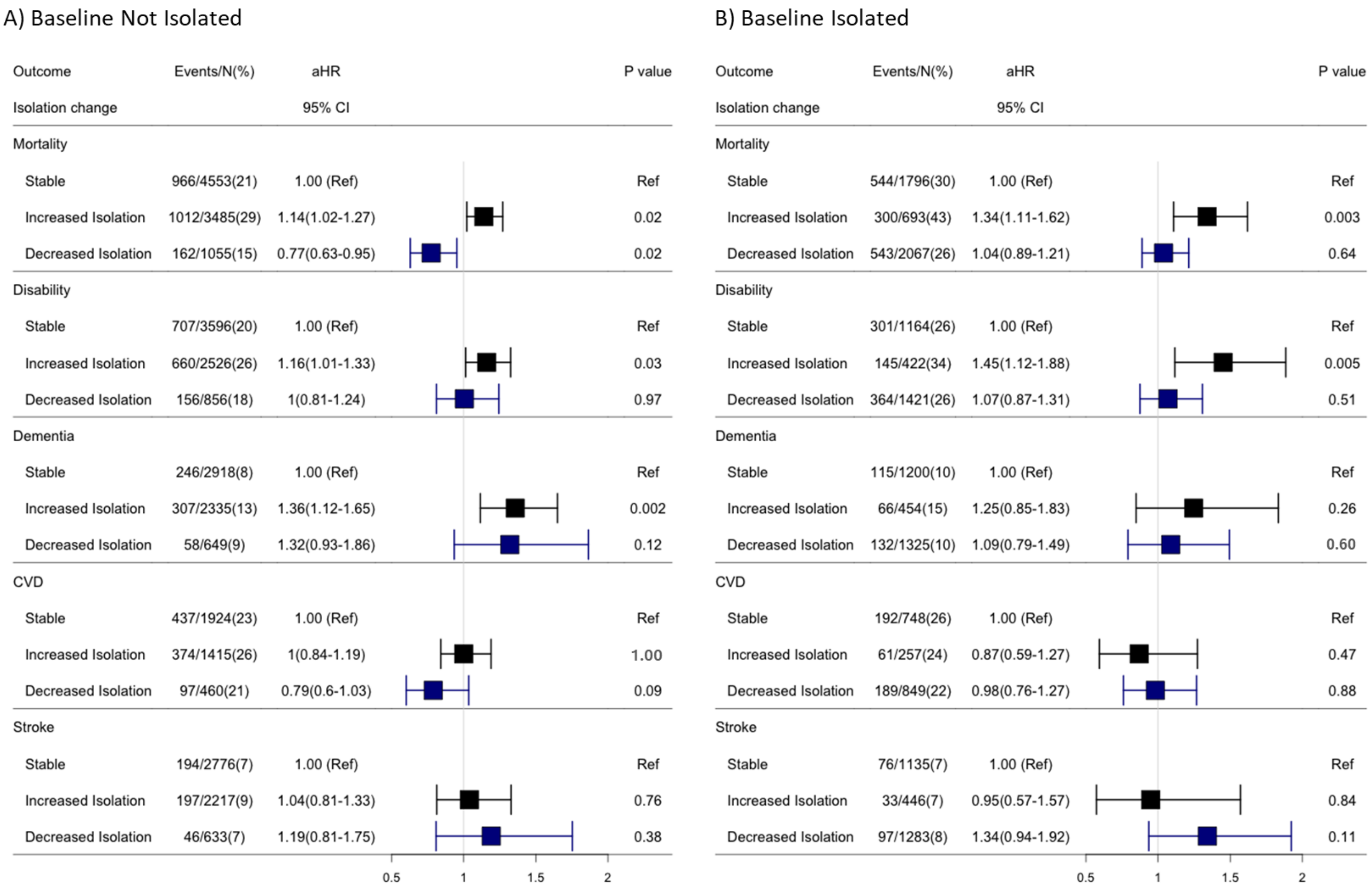

© 2024 Lyu C et al. *JAMA Network Open.*

Etable 1: ICD-10 Diagnosis codes from Medicare Chronic Conditions Warehouse.

Outcome	ICD-10 Codes
Atrial fibrillation	I48.0, I48.1, I48.11, I48.19, I48.2, I48.20, I48.21, I48.3, I48.4, I48.91
Congestive heart failure	I09.81, I11.0, I13.0, I13.2, I42.0, I42.5, I42.6, I42.7, I42.8, I43, I50.1, I50.20, I50.21, I50.22, I50.23, I50.30, I50.31, I50.32, I50.33, I50.40, I50.41, I50.42, I50.43, I50.810, I50.811, I50.812, I50.813, I50.814, I50.82, I50.83, I50.84, I50.89, I50.9, P29.0
Ischemic heart disease	I20.0, I20.1, I20.8, I24.0, I24.1, I24.8, I25.10, I25.110, I25.111, I25.118, I25.119, I25.3, I25.41, I25.42, I25.5, I25.6, I25.700, I25.701, I25.708, I25.710, I25.711, I25.718, I25.719, I25.720, I25.721, I25.728, I25.729, I25.730, I25.731, I25.738, I25.739, I25.750, I25.751, I25.758, I25.759, I25.760, I25.761, I25.768, I25.769, I25.790, I25.791, I25.798, I25.799, I25.810, I25.811, I25.812, I25.82, I25.83, I25.84, I25.89, I25.9
Acute myocardial infarction	I21.01, I21.02, I21.09, I21.11, I21.19, I21.21, I21.29, I21.3, I21.4, I21.9, I21.A1, I21.A9, I22.0, I22.1, I22.2, I22.8, I22.9
Stroke*	G45.0, G45.1, G45.2, G45.3, G45.8, G45.9, G46.0, G46.1, G46.2, G46.3, G46.4, G46.5, G46.6, G46.7, G46.8, G97.31, G97.32, I60.00, I60.01, I60.02, I60.10, I60.11, I60.12, I60.2, I60.20, I60.21, I60.22, I60.30, I60.31, I60.32, I60.4, I60.50, I60.51, I60.52, I60.6, I60.7, I60.8, I60.9, I61.0, I61.1, I61.2, I61.3, I61.4, I61.5, I61.6, I61.8, I61.9, I62.00, I62.01, I62.02, I62.9, I63.00, I63.011, I63.012, I63.013, I63.019, I63.02, I63.031, I63.032, I63.033, I63.039, I63.09, I63.10, I63.111, I63.112, I63.113, I63.119, I63.12, I63.131, I63.132, I63.133, I63.139, I63.19, I63.20, I63.211, I63.212, I63.213, I63.219, I63.22, I63.231, I63.232, I63.233, I63.239, I63.29, I63.30, I63.311, I63.312, I63.313, I63.319, I63.321, I63.322, I63.323, I63.329, I63.331, I63.332, I63.333, I63.339, I63.341, I63.342, I63.343, I63.349, I63.39, I63.40, I63.411, I63.412, I63.413, I63.419, I63.421, I63.422, I63.423, I63.429, I63.431, I63.432, I63.433, I63.439, I63.441, I63.442, I63.443, I63.449, I63.49, I63.50, I63.511, I63.512, I63.513, I63.519, I63.521, I63.522, I63.523, I63.529, I63.531, I63.532, I63.533, I63.539, I63.541, I63.542, I63.543, I63.549,

© 2024 Lyu C et al. *JAMA Network Open.*

	I63.59, I63.6, I63.8, I63.81, I63.89, I63.9, I67.841, I67.848, I67.89, I97.810, I97.811, I97.820, I97.821
Alzheimer's Disease	G30.0, G30.1, G30.8, G30.9
Alzheimer's Disease and Related Disorders or Senile Dementia	F01.50, F01.51, F02.80, F02.81, F03.90, F03.91, F04, F05, F06.1, F06.8, G13.8, G30.0, G30.1, G30.8, G30.9, G31.01, G31.09, G31.1, G31.2, G94, R41.81, R54

** If any of the qualifying claims have any of the following codes in any DX position then Stroke diagnosis was excluded: S06.340A, S06.341A, S06.350A, S06.351A, S06.360A, S06.361A, S06.370A, S06.371A, S06.380A, S06.381A, S06.5X0A, S06.5X1A, S06.6X0A, S06.6X1A, S06.810A, S06.811A, S06.820A, S06.821A, S06.890A, S06.891A, S06.9X0A, S06.9X1A, S06.A0XA, S06.A1XA, S06.342A, S06.343A, S06.344A, S06.345A, S06.346A, S06.347A, S06.348A, S06.349A, S06.352A, S06.353A, S06.354A, S06.355A, S06.356A, S06.357A, S06.358A, S06.359A, S06.362A, S06.363A, S06.364A, S06.365A, S06.366A, S06.367A, S06.368A, S06.369A, S06.372A, S06.373A, S06.374A, S06.375A, S06.376A, S06.377A, S06.378A, S06.379A, S06.382A, S06.383A, S06.384A, S06.385A, S06.386A, S06.387A, S06.388A, S06.389A, S06.5X2A, S06.5X3A, S06.5X4A, S06.5X5A, S06.5X6A, S06.5X7A, S06.5X8A, S06.5X9A, S06.6X2A, S06.6X3A, S06.6X4A, S06.6X5A, S06.6X6A, S06.6X7A, S06.6X8A, S06.6X9A, S06.812A, S06.813A, S06.814A, S06.815A, S06.816A, S06.817A, S06.818A, S06.819A, S06.822A, S06.823A, S06.824A, S06.825A, S06.826A, S06.827A, S06.828A, S06.829A, S06.892A, S06.893A, S06.894A, S06.895A, S06.896A, S06.897A, S06.898A, S06.899A, S06.9X2A, S06.9X3A, S06.9X4A, S06.9X5A, S06.9X6A, S06.9X7A, S06.9X8A, S06.9X9A*

© 2024 Lyu C et al. *JAMA Network Open.*

Etable 2: Social Isolation Index change from baseline to second social isolation measurement stratified by isolation status

	Baseline Non-isolation	Baseline Isolation
Decreased isolation, mean (SD)	-1.00 (0)	-1.37 (0.63)
Stable, mean (SD)	0 (0)	0 (0)
Increased isolation, mean (SD)	1.30 (0.62)	1.41 (0.60)

© 2024 Lyu C et al. *JAMA Network Open.*

Etable 3: Demographic and clinical characteristics of the HRS analysis cohort by social isolation group from baseline to second social isolation measure.

A) Demographic characteristics of the HRS analysis cohort with **baseline not isolated** and by social isolation group from baseline to second social isolation measure

	Decreased Isolation, No. (%)	Stable, No. (%)	Increased Isolation, No. (%)	*P* value[a]
**Patients, No.**	1055	4553	3485	
**Sex**				<0.001
**Male**	513 (48.6)	1906 (41.9)	1374 (39.4)	
**Female**	542 (51.4)	2647 (58.1)	2111 (60.6)	
**Cohort**[b]				<0.001
**AHEAD**	16 (1.5)	135 (3.0)	174 (5.0)	
**CODA**	81 (7.7)	404 (8.9)	452 (13.0)	
**HRS**	344 (32.6)	1566 (34.4)	1221 (35.0)	
**WB**	185 (17.5)	771 (16.9)	497 (14.3)	
**BB**	429 (40.7)	1677 (36.8)	1141 (32.7)	
**Race and ethnicity**[c]				<0.001
**Hispanic**	93 (8.8)	365 (8.0)	345 (9.9)	
**Non-Hispanic Black**	121 (11.5)	598 (13.1)	535 (15.4)	
**Non-Hispanic White**	811 (76.9)	3482 (76.5)	2517 (72.2)	
**Other**	30 (2.8)	108 (2.4)	88 (2.5)	
**Educational Level, y**				<0.001
**<12**	122 (11.6)	640 (14.1)	650 (18.7)	
**12**	329 (31.2)	1496 (32.9)	1185 (34.0)	

© 2024 Lyu C et al. *JAMA Network Open.*

**>12**	604 (57.3)	2417 (53.1)	1650 (47.3)	
**Baseline characteristics**				
**Age, mean (SD), y**	63.54 (8.67)	64.65 (9.09)	66.30 (9.77)	<0.001
**Proxy vs Self**	1043 (98.9)	4499 (98.8)	3446 (98.9)	0.96
**Baseline Year**				0.21
**2006**	449 (42.6)	1839 (40.4)	1447 (41.5)	
**2008**	337 (31.9)	1586 (34.8)	1225 (35.2)	
**2010**	138 (13.1)	561 (12.3)	383 (11.0)	
**2012**	107 (10.1)	466 (10.2)	336 (9.6)	
**2014**	24 (2.3)	101 (2.2)	94 (2.7)	
**BMI, mean (SD)[d]**	28.36 (5.55)	28.54 (5.75)	28.57 (5.70)	0.56
**Hypertension**	484 (45.9)	2327 (51.1)	1892 (54.3)	<0.001
**Diabetes**	156 (14.8)	755 (16.6)	637 (18.3)	0.02
**Lung Disease**	52 (4.9)	257 (5.6)	254 (7.3)	0.002
**Heart Disease**	181 (17.2)	785 (17.2)	707 (20.3)	0.001
**Stroke**	54 (5.1)	210 (4.6)	195 (5.6)	0.14
**Cancer**	120 (11.4)	554 (12.2)	466 (13.4)	0.15
**Psychiatric problems**	103 (9.8)	498 (10.9)	462 (13.3)	0.13
**Arthritis**	499 (47.3)	2397 (52.6)	1964 (56.4)	0.001
**Total Assets, quantile**				<0.001
**1st**	129 (12.2)	543 (11.9)	533 (15.3)	
**2nd**	159 (15.1)	779 (17.1)	737 (21.1)	
**3rd**	260 (24.6)	1181 (25.9)	959 (27.5)	
**4th**	507 (48.1)	2050 (45.0)	1256 (36.0)	
**CES-D score, mean (SD)**	0.91 (1.51)	1.01 (1.60)	1.28 (1.84)	<0.001
**Current Smoker**	101 (9.6)	508 (11.2)	437 (12.5)	0.02
**Married vs not married**	100 (9.5)	735 (16.1)	820 (23.5)	<0.001

© 2024 Lyu C et al. *JAMA Network Open.*

**ADL score, mean (SD)[e]**	0.15 (0.58)	0.16 (0.59)	0.24 (0.77)	<0.001
**Cognition, mean (SD)**	16.61 (3.74)	16.32 (3.80)	15.58 (3.98)	<0.001
**Incidence Disease from Baseline to Second Social Isolation Measure**				
**Hypertension**	86 (8.2)	357 (7.8)	274 (7.9)	0.94
**Diabetes**	73 (6.9)	259 (5.7)	188 (5.4)	0.17
**Lung Disease**	23 (2.2)	111 (2.4)	104 (3.0)	0.20
**Heart Disease**	63 (6.0)	282 (6.2)	243 (7.0)	0.29
**Stroke**	25 (2.4)	102 (2.2)	137 (3.9)	<0.001
**Cancer**	40 (3.8)	183 (4.0)	151 (4.3)	0.67
**Psychiatric Problem**	25 (2.4)	128 (2.8)	133 (3.8)	0.01
**Arthritis**	85 (8.1)	332 (7.3)	277 (7.9)	0.47
**Change from Baseline to Second Social Isolation Measure, mean (SD)**				
**CES-D score**	-0.02 (1.37)	0.02 (1.40)	0.09 (1.57)	0.03
**ADL score[e]**	0.01 (0.55)	0.04 (0.57)	0.10 (0.72)	<0.001
**Cognition**	-0.36 (2.98)	-0.37 (2.91)	-0.54 (3.07)	0.03
**Follow up years for each outcome, median (range)**				
**Mortality**	8 (0.5-10)	8 (0.1-10)	8 (0.3-10)	
**Disability**	8 (0-10)	8 (0-10)	8 (0-10)	
**Dementia**	7 (0.1-7)	7 (0-7)	7 (0-7)	
**CVD**	7 (0.1-7)	7 (0-7)	7 (0-7)	
**Stroke**	7 (0.1-7)	7 (0.1-7)	7 (0.1-7)	

© 2024 Lyu C et al. *JAMA Network Open.*

B) Demographic characteristics of the HRS analysis cohort with baseline isolated and by social isolation group from baseline to second social isolation measure

	Decreased Isolation, No. (%)	Stable, No. (%)	Increased Isolation, No. (%)	*P* value[a]
**Patients, No.**	2067	1796	693	
**Sex**				<0.001
**Male**	919 (44.5)	631 (35.1)	295 (42.6)	
**Female**	1148 (55.5)	1165 (64.9)	398 (57.4)	
**Cohort[b]**				<0.001
**AHEAD**	82 (4.0)	101 (5.6)	67 (9.7)	
**CODA**	183 (8.9)	193 (10.7)	101 (14.6)	
**HRS**	679 (32.8)	612 (34.1)	215 (31.0)	
**WB**	289 (14.0)	236 (13.1)	81 (11.7)	
**BB**	834 (40.3)	654 (36.4)	229 (33.0)	
**Race and ethnicity[c]**				0.07
**Hispanic**	256 (12.4)	194 (10.8)	79 (11.4)	
**Non-Hispanic Black**	405 (19.6)	305 (17.0)	110 (15.9)	
**Non-Hispanic White**	1342 (64.9)	1245 (69.3)	486 (70.1)	
**Other**	64 (3.1)	52 (2.9)	18 (2.6)	
**Educational level**				0.003
**<12**	477 (23.1)	452 (25.2)	203 (29.3)	
**12**	697 (33.7)	635 (35.4)	238 (34.3)	
**>12**	893 (43.2)	709 (39.5)	252 (36.4)	
**Baseline characteristics**				
**Age, mean (SD)**	64.80 (9.53)	65.85 (9.84)	67.88 (10.80)	<0.001
**Proxy vs self**	2043 (98.8)	1767 (98.4)	683 (98.6)	0.48

© 2024 Lyu C et al. *JAMA Network Open.*

**Baseline Year**				0.27
**2006**	787 (38.1)	659 (36.7)	260 (37.5)	
**2008**	695 (33.6)	673 (37.5)	264 (38.1)	
**2010**	294 (14.2)	235 (13.1)	87 (12.6)	
**2012**	234 (11.3)	189 (10.5)	67 (9.7)	
**2014**	57 (2.8)	40 (2.2)	15 (2.2)	
**BMI, mean (SD)[d]**	29.00 (6.08)	28.99 (6.63)	28.43 (6.51)	0.10
**Hypertension**	1139 (55.1)	1018 (56.7)	398 (57.4)	0.46
**Diabetes**	431 (20.9)	365 (20.3)	148 (21.4)	0.83
**Lung disease**	188 (9.1)	181 (10.1)	71 (10.2)	0.50
**Heart disease**	394 (19.1)	370 (20.6)	167 (24.1)	0.02
**Stroke**	132 (6.4)	129 (7.2)	67 (9.7)	0.02
**Cancer**	258 (12.5)	205 (11.4)	77 (11.1)	0.48
**Psychiatric problems**	363 (17.6)	327 (18.2)	148 (21.4)	0.08
**Arthritis**	1110 (53.7)	1056 (58.8)	385 (55.6)	0.006
**Total assets, quantile**				0.001
**1st**	584 (28.3)	549 (30.6)	242 (34.9)	
**2nd**	478 (23.1)	456 (25.4)	173 (25.0)	
**3rd**	473 (22.9)	396 (22.0)	142 (20.5)	
**4th**	532 (25.7)	395 (22.0)	136 (19.6)	
**CES-D score, mean (SD)**	1.71 (2.16)	1.85 (2.25)	2.18 (2.35)	<0.001
**Current Smoker**	336 (16.3)	343 (19.1)	145 (20.9)	0.008
**Married vs not married**	949 (45.9)	1182 (65.8)	449 (64.8)	<0.001
**ADL score, mean (SD)[e]**	0.34 (0.94)	0.39 (1.00)	0.51 (1.13)	<0.001
**Cognition, mean (SD)**	15.08 (4.17)	15.27 (4.09)	14.14 (4.41)	<0.001
**Incidence Disease from Baseline to Second Social Isolation Measure**				
**Hypertension**	177 (8.6)	149 (8.3)	65 (9.4)	0.69
**Diabetes**	104 (5.0)	104 (5.8)	34 (4.9)	0.50

© 2024 Lyu C et al. *JAMA Network Open.*

**Lung disease**	70 (3.4)	88 (4.9)	34 (4.9)	0.04
**Heart disease**	128 (6.2)	127 (7.1)	50 (7.2)	0.46
**Stroke**	61 (3.0)	51 (2.8)	30 (4.3)	0.13
**Cancer**	90 (4.4)	67 (3.7)	28 (4.0)	0.62
**Psychiatric problem**	67 (3.2)	59 (3.3)	45 (6.5)	<0.001
**Arthritis**	163 (7.9)	139 (7.7)	57 (8.2)	0.92
**Change from Baseline to Second Social Isolation Measure, mean (SD)**				
**CESD score**	-0.13 (1.74)	-0.10 (1.68)	-0.06 (1.97)	0.60
**ADL score[e]**	0.07 (0.78)	0.10 (0.83)	0.26 (1.05)	<0.001
**Cognition**	-0.47 (3.14)	-0.58 (3.06)	-0.79 (3.21)	0.06
**Follow up years for each outcome, median (range)**				
**Mortality**	8 (0.5-10)	8 (0.4-10)	8 (0.3-10)	
**Disability**	8 (0-10)	8 (0-10)	8 (0-10)	
**Dementia**	7 (0-7)	7 (0.2-7)	7 (0-7)	
**CVD**	7 (0-7)	7 (0-7)	7 (0.1-7)	
**Stroke**	7 (0-7)	7 (0.1-7)	7 (0-7)	

a. P-values are from the two-sample t-test or Wilcoxon rank sum test for comparing continuous covariates and the chi-square test for comparing categorical covariates; p value <0.05 was considered as significant and marked in red color.
b. Other race includes American Indian, Alaskan Native, Asian, Native Hawaiian, and Pacific Islander, other race or unknown.
c. HRS included six birth cohorts: the Study of Assets and Health Dynamics Among the Oldest Old (AHEAD) cohort, born prior to1924; the Children of the Depression (CODA) cohort, born1924 to1930; the original HRS cohort, born1931 to1941; the War Baby (WB) cohort, born1942 to1947; the Baby Boomer (BB) cohort, born1948 to1959.
d. BMI: body mass index
e. ADL: activities of daily living
f. Model-adjusted 27-point cognition score

© 2024 Lyu C et al. *JAMA Network Open.*

Etable 4: The hazard ratios of full covariates on distal outcomes stratified by the social isolation status

A) The hazard ratios of full covariates on mortality stratified by the social isolation status

	**Baseline Non-isolation**		**Baseline Isolation**	
	aHR (95% CI)	*P* value	aHR (95% CI)	*P* value
Baseline isolation (ref: baseline non-isolation)	0.98 (0.84, 1.14)	0.75	1.03 (0.88, 1.19)	0.75
Increased isolation (ref: stable)	1.10 (1.01, 1.21)	0.04	1.29 (1.09, 1.51)	0.002
Decreased isolation (ref: stable)	0.73 (0.61, 0.87)	<0.001	0.98 (0.86, 1.12)	0.78
Sex = female (ref: male)	0.75 (0.70, 0.82)	<0.001	0.75 (0.70, 0.82)	<0.001
Cohort = CODA (ref: AHEAD)	1.14 (0.98, 1.34)	0.09	1.14 (0.98, 1.34)	0.09
Cohort = HRS (ref: AHEAD)	1.33 (1.06, 1.67)	0.01	1.33 (1.06, 1.67)	0.01
Cohort = WB (ref: AHEAD)	1.56 (1.12, 2.19)	0.01	1.56 (1.12, 2.19)	0.009
Cohort = BB (ref: AHEAD)	2.16 (1.43, 3.27)	<0.001	2.16 (1.43, 3.27)	<0.001
Race and ethnicity = Non-Hispanic Black (ref: Hispanic)	1.33 (1.10, 1.60)	0.004	1.33 (1.10, 1.60)	0.004
Race and ethnicity = Non-Hispanic White (ref: Hispanic)	1.64 (1.39, 1.94)	<0.001	1.64 (1.39, 1.94)	<0.001
Race and ethnicity = Other (ref: Hispanic)	1.14 (0.79, 1.63)	0.48	1.14 (0.79, 1.63)	0.48
Educational level = 12 (ref: < 12)	0.99 (0.90, 1.10)	0.89	0.99 (0.90, 1.10)	0.89
Educational level > 12 (ref: < 12)	1.00 (0.89, 1.11)	0.93	1.00 (0.89, 1.11)	0.93
Age at baseline	1.12 (1.11, 1.13)	<0.001	1.12 (1.11, 1.13)	<0.001
BMI at baseline	1.00 (1.00, 1.01)	0.35	1.00 (1.00, 1.011)	0.35
Hypertension at baseline	1.15 (1.06, 1.25)	0.001	1.15 (1.06, 1.25)	0.001
Diabetes at baseline	1.34 (1.22, 1.46)	<0.001	1.34 (1.22, 1.46)	<0.001
Lung disease at baseline	1.63 (1.44, 1.85)	<0.001	1.63 (1.44, 1.85)	<0.001
Heart disease at baseline	1.42 (1.30, 1.54)	<0.001	1.42 (1.30, 1.54)	<0.001
Stroke at baseline	1.34 (1.18, 1.53)	<0.001	1.34 (1.18, 1.53)	<0.001
Cancer at baseline	1.48 (1.35, 1.62)	<0.001	1.48 (1.35, 1.62)	<0.001
Psychiatric problems at baseline	0.96 (0.85, 1.09)	0.57	0.96 (0.85, 1.09)	0.57
Arthritis at baseline	0.97 (0.90, 1.06)	0.53	0.97 (0.90, 1.06)	0.53
CES-D score at baseline	1.06 (1.03, 1.08)	<0.001	1.06 (1.03, 1.08)	<0.001
Smoker at baseline	1.91 (1.71, 2.14)	<0.001	1.91 (1.71, 2.14)	<0.001
Married vs not married at baseline =not married (ref: married)	0.98 (0.89, 1.08)	0.66	0.98 (0.89, 1.08)	0.66
ADL at baseline	1.03 (0.98, 1.09)	0.22	1.03 (0.98, 1.09)	0.22
Total assets at baseline = 2nd quantile (ref: 1st quantile)	1.00 (0.89, 1.13)	0.97	1.00 (0.89, 1.13)	0.97

© 2024 Lyu C et al. *JAMA Network Open.*

Total assets at baseline = 3rd quantile (ref: 1st quantile)	0.84 (0.74, 0.95)	0.006	0.84 (0.74, 0.95)	0.006
Total assets at baseline = 4th quantile (ref: 1st quantile)	0.80 (0.71, 0.91)	0.001	0.80 (0.71, 0.91)	0.001
Cognition at baseline	0.96 (0.95, 0.97)	<0.001	0.96 (0.95, 0.97)	<0.001
Social isolation at baseline	1.07 (0.99, 1.14)	0.08	1.07 (0.99, 1.14)	0.08
Incident hypertension from baseline to second social isolation measure	1.02 (0.88, 1.17)	0.82	1.02 (0.88, 1.17)	0.82
Incident diabetes from baseline to second social isolation measure	1.04 (0.88, 1.22)	0.64	1.04 (0.88, 1.22)	0.64
Incident lung disease from baseline to second social isolation measure	1.93 (1.66, 2.26)	<0.001	1.93 (1.66, 2.26)	<0.001
Incident heart disease from baseline to second social isolation measure	1.37 (1.20, 1.56)	<0.001	1.37 (1.20, 1.56)	<0.001
Incident stroke from baseline to second social isolation measure	1.53 (1.29, 1.81)	<0.001	1.53 (1.29, 1.81)	<0.001
Incident cancer from baseline to second social isolation measure	1.78 (1.50, 2.10)	<0.001	1.78 (1.50, 2.10)	<0.001
Incident psychiatric problems from baseline to second social isolation measure	1.06 (0.87, 1.29)	0.56	1.06 (0.87, 1.29)	0.56
Incident arthritis from baseline to second social isolation measure	0.94 (0.80, 1.09)	0.40	0.94 (0.80, 1.09)	0.40
CES-D score change from baseline to second social isolation measure	1.07 (1.04, 1.10)	<0.001	1.07 (1.04, 1.10)	<0.001
Baseline isolation: increased isolation	1.16 (0.97, 1.40)	0.11	0.86 (0.71, 1.03)	0.11
Baseline isolation: decreased isolation	1.35 (1.08, 1.67)	0.008	0.74 (0.60, 0.93)	0.008

© 2024 Lyu C et al. *JAMA Network Open.*

B) The hazard ratios of full covariates on disability stratified by the social isolation status

	Baseline Non-isolation		Baseline Isolation	
	aHR (95% CI)	*P* value	aHR (95% CI)	*P* value
Baseline isolation (ref: baseline non-isolation)	1.02 (0.85, 1.23)	0.83	0.98 (0.81, 1.18)	0.83
Increased isolation (ref: stable)	1.15 (1.03, 1.28)	0.01	1.35 (1.09, 1.68)	0.006
Decreased isolation (ref: stable)	1.04 (0.88, 1.25)	0.63	1.05 (0.88, 1.24)	0.59
Sex=female (ref: male)	1.00 (0.91, 1.10)	0.94	1.00 (0.91, 1.10)	0.94
Cohort = CODA (ref: AHEAD)	0.92 (0.73, 1.16)	0.48	0.92 (0.73, 1.16)	0.48
Cohort = HRS (ref: AHEAD)	0.83 (0.62, 1.11)	0.21	0.83 (0.62, 1.11)	0.21
Cohort = WB (ref: AHEAD)	0.73 (0.49, 1.08)	0.12	0.73 (0.49, 1.08)	0.12
Cohort = BB (ref: AHEAD)	0.77 (0.48, 1.23)	0.27	0.77 (0.48, 1.23)	0.27
Race and ethnicity = Non-Hispanic Black (ref: Hispanic)	0.84 (0.70, 1.02)	0.08	0.84 (0.70, 1.02)	0.08
Race and ethnicity = Non-Hispanic White (ref: Hispanic)	0.84 (0.71, 0.99)	0.04	0.84 (0.71, 0.99)	0.04
Race and ethnicity = Other (ref: Hispanic)	0.78 (0.56, 1.10)	0.15	0.78 (0.56, 1.10)	0.15
Educational level = 12 (ref: < 12)	0.97 (0.85, 1.10)	0.64	0.97 (0.85, 1.10)	0.64
Educational level > 12 (ref: < 12)	1.02 (0.89, 1.16)	0.79	1.02 (0.89, 1.16)	0.79
Age at baseline	1.05 (1.03, 1.06)	<0.001	1.05 (1.03, 1.06)	<0.001
BMI at baseline	1.04 (1.03, 1.05)	<0.001	1.04 (1.03, 1.05)	<0.001
Hypertension at baseline	0.97 (0.88, 1.07)	0.59	0.97 (0.88, 1.07)	0.59
Diabetes at baseline	1.20 (1.07, 1.34)	0.002	1.20 (1.07, 1.34)	0.002
Lung disease at baseline	1.20 (1.01, 1.42)	0.04	1.20 (1.01, 1.42)	0.04
Heart disease at baseline	1.12 (1.00, 1.26)	0.04	1.12 (1.00, 1.26)	0.04
Stroke at baseline	1.16 (0.95, 1.42)	0.15	1.16 (0.95, 1.42)	0.15
Cancer at baseline	1.09 (0.97, 1.24)	0.16	1.09 (0.97, 1.4)	0.16
Psychiatric problems at baseline	1.09 (0.94, 1.26)	0.27	1.09 (0.94, 1.26)	0.27
Arthritis at baseline	1.57 (1.42, 1.73)	<0.001	1.57 (1.42, 1.73)	<0.001
CES-D score at baseline	1.23 (1.19, 1.27)	<0.001	1.23 (1.19, 1.27)	<0.001
Smoker at baseline	1.36 (1.19, 1.55)	<0.001	1.36 (1.19, 1.55)	<0.001
Married vs not married at baseline = not married (ref: married)	0.98 (0.88, 1.10)	0.74	0.98 (0.88, 1.10)	0.74
Total assets at baseline = 2nd quantile (ref: 1st quantile)	0.92 (0.80, 1.06)	0.26	0.92 (0.80, 1.06)	0.26
Total assets at baseline = 3rd quantile (ref: 1st quantile)	0.72 (0.62, 0.83)	<0.001	0.72 (0.62, 0.83)	<0.001
Total assets at baseline = 4th quantile (ref: 1st quantile)	0.72 (0.61, 0.83)	<0.001	0.72 (0.61, 0.83)	<0.001
Cognition at baseline	0.98 (0.97, 1.00)	0.01	0.98 (0.97, 1.00)	0.01

© 2024 Lyu C et al. *JAMA Network Open.*

Social isolation at baseline	1.03 (0.94, 1.12)	0.53	1.03 (0.94, 1.12)	0.53
Incident hypertension from baseline to second social isolation measure	1.14 (0.98, 1.34)	0.10	1.14 (0.98, 1.34)	0.10
Incident diabetes from baseline to second social isolation measure	0.96 (0.78, 1.17)	0.67	0.96 (0.78, 1.17)	0.67
Incident lung disease from baseline to second social isolation measure	1.28 (1.01, 1.63)	0.05	1.28 (1.01, 1.63)	0.05
Incident heart disease from baseline to second social isolation measure	1.17 (0.99, 1.38)	0.07	1.17 (0.99, 1.38)	0.07
Incident stroke from baseline to second social isolation measure	1.45 (1.14, 1.83)	0.002	1.45 (1.14, 1.83)	0.002
Incident cancer from baseline to second social isolation measure	1.07 (0.86, 1.33)	0.52	1.07 (0.86, 1.33)	0.52
Incident psychiatric problems from baseline to second social isolation measure	1.32 (1.03, 1.68)	0.03	1.32 (1.03, 1.68)	0.03
Incident arthritis from baseline to second social isolation measure	1.70 (1.46, 1.99)	<0.001	1.70 (1.46, 1.99)	<0.001
CES-D score change from baseline to second social isolation measure	1.20 (1.16, 1.24)	<0.001	1.20 (1.16, 1.24)	<0.001
Baseline isolation: increased isolation	1.18 (0.924, 1.50)	0.19	0.85 (0.67, 1.08)	0.19
Baseline isolation: decreased isolation	1.00 (0.789, 1.28)	0.98	0.997 (0.78, 1.27)	0.98

© 2024 Lyu C et al. *JAMA Network Open.*

C) The hazard ratios of full covariates on dementia stratified by the social isolation status

	**Baseline Non-isolation**		**Baseline Isolation**	
	aHR (95% CI)	*P* value	aHR (95% CI)	*P* value
Baseline isolation (ref: baseline non-isolation)	0.85 (0.63, 1.14)	0.28	1.18 (0.87, 1.59)	0.28
Increased isolation (ref: stable)	1.29 (1.08, 1.54)	0.004	1.40 (1.02, 1.93)	0.04
Decreased isolation (ref: stable)	1.27 (0.94, 1.71)	0.12	1.27 (0.96, 1.68)	0.09
Sex = female (ref: male)	1.27 (1.09, 1.48)	0.002	1.27 (1.09, 1.48)	0.002
Cohort = CODA (ref: AHEAD)	1.14 (0.86, 1.50)	0.37	1.14 (0.86, 1.50)	0.37
Cohort = HRS (ref: AHEAD)	1.08 (0.71, 1.64)	0.71	1.08 (0.71, 1.64)	0.71
Cohort = WB (ref: AHEAD)	0.47 (0.25, 0.88)	0.02	0.47 (0.25, 0.88)	0.02
Cohort = BB (ref: AHEAD)	0.55 (0.26, 1.16)	0.12	0.55 (0.26, 1.16)	0.12
Race and ethnicity = Non-Hispanic Black (ref: Hispanic)	1.73 (1.21, 2.47)	0.003	1.73 (1.21, 2.47)	0.003
Race and ethnicity = Non-Hispanic White (ref: Hispanic)	1.66 (1.20, 2.31)	0.002	1.66 (1.20, 2.31)	0.002
Race and ethnicity = Other (ref: Hispanic)	1.42 (0.76, 2.63)	0.27	1.42 (0.76, 2.63)	0.27
Educational level = 12 (ref: < 12)	1.20 (0.99, 1.46)	0.06	1.20 (0.99, 1.46)	0.06
Educational level > 12 (ref: < 12)	1.30 (1.05, 1.60)	0.02	1.30 (1.05, 1.60)	0.02
Age at baseline	1.07 (1.05, 1.10)	<0.001	1.07 (1.05, 1.10)	<0.001
BMI at baseline	0.98 (0.96, 0.99)	0.001	0.98 (0.96, 0.99)	0.001
Hypertension at baseline	1.01 (0.86, 1.18)	0.91	1.01 (0.86, 1.18)	0.91
Diabetes at baseline	1.34 (1.13, 1.59)	0.001	1.34 (1.13, 1.59)	0.001
Lung disease at baseline	0.88 (0.70, 1.12)	0.31	0.88 (0.70, 1.12)	0.31
Heart disease at baseline	1.00 (0.84, 1.17)	0.95	1.00 (0.84, 1.17)	0.95
Stroke at baseline	1.46 (1.16, 1.83)	0.001	1.46 (1.16, 1.83)	0.001
Cancer at baseline	0.90 (0.75, 1.08)	0.25	0.90 (0.75, 1.08)	0.25
Psychiatric problems at baseline	1.48 (1.20, 1.83)	<0.001	1.48 (1.20, 1.83)	<0.001
Arthritis at baseline	1.02 (0.87, 1.21)	0.78	1.02 (0.87, 1.21)	0.78
CES-D score at baseline	1.10 (1.05, 1.15)	<0.001	1.10 (1.05, 1.15)	<0.001
Smoker at baseline	1.36 (1.08, 1.71)	0.009	1.36 (1.08, 1.71)	0.009
Married vs not married at baseline = not married (ref: married)	0.84 (0.70, 1.00)	0.05	0.84 (0.70, 1.00)	0.05
ADL at baseline	1.09 (1.00, 1.18)	0.04	1.09 (1.00, 1.18)	0.04
Total assets at baseline = 2nd quantile (ref: 1st quantile)	1.05 (0.83, 1.34)	0.67	1.05 (0.83, 1.34)	0.67
Total assets at baseline = 3rd Quantile (ref: 1st quantile)	0.93 (0.73, 1.18)	0.54	0.93 (0.73, 1.18)	0.54
Total assets at baseline = 4th Quantile (ref: 1st quantile)	0.89 (0.69, 1.15)	0.39	0.89 (0.69, 1.15)	0.39

© 2024 Lyu C et al. *JAMA Network Open.*

Cognition at baseline	0.92 (0.90, 0.94)	<0.001	0.92 (0.90, 0.94)	<0.001
Social isolation at baseline	1.02 (0.88, 1.17)	0.80	1.02 (0.88, 1.17)	0.80
Incident hypertension from baseline to second social isolation measure	1.04 (0.79, 1.37)	0.78	1.04 (0.79, 1.37)	0.78
Incident diabetes from baseline to second social isolation measure	1.11 (0.81, 1.52)	0.50	1.11 (0.81, 1.52)	0.50
Incident lung disease from baseline to second social isolation measure	0.59 (0.37, 0.92)	0.02	0.59 (0.37, 0.92)	0.02
Incident heart disease from baseline to second social isolation measure	1.08 (0.85, 1.38)	0.51	1.08 (0.85, 1.38)	0.51
Incident stroke from baseline to second social isolation measure	1.49 (1.09, 2.02)	0.01	1.49 (1.09, 2.02)	0.01
Incident cancer from baseline to second social isolation measure	1.08 (0.78, 1.49)	0.64	1.08 (0.78, 1.49)	0.64
Incident psychiatric problems from baseline to second social isolation measure	1.61 (1.12, 2.32)	0.01	1.61 (1.12, 2.32)	0.01
Incident arthritis from baseline to second social isolation measure	1.08 (0.80, 1.46)	0.61	1.08 (0.80, 1.46)	0.61
CES-D score change from baseline to second social isolation measure	1.084 (1.026, 1.144)	0.004	1.08 (1.03, 1.14)	0.004
Baseline isolation: increased isolation	1.084 (0.751, 1.567)	0.67	0.92 (0.64, 1.33)	0.67
Baseline isolation: decreased isolation	1.001 (0.672, 1.491)	1.00	1.00 (0.67, 1.49)	1.00

© 2024 Lyu C et al. *JAMA Network Open.*
