# Supplementary material for: Social Isolation Changes and Long-Term Outcomes Among Older Adults
Source: JAMA Netw Open. 2024 Jul 24;7(7):e2424519. doi: 10.1001/jamanetworkopen.2024.24519 (PMC11270134; doi:10.1001/jamanetworkopen.2024.24519)
Supplement: Supplement 2. — Data Sharing Statement [file jamanetwopen-e2424519-s002.pdf]

Data Sharing Statement

Lyu. Social Isolation Changes and Long-Term Outcomes Among Older Adults. *JAMA Netw Open*. Published July 24, 2024. doi:10.1001/jamanetworkopen.2024.24519

Data
Data available: No

Additional Information
Explanation for why data not available: The Health and Retirement Study (HRS) is partly public available through their website, the restricted HRS data can be obtained by application.
